# IL-6 in the infarcted heart is preferentially formed by fibroblasts and modulated by purinergic signaling

**DOI:** 10.1172/JCI163799

**Published:** 2023-06-01

**Authors:** Christina Alter, Anne-Sophie Henseler, Christoph Owenier, Julia Hesse, Zhaoping Ding, Tobias Lautwein, Jasmin Bahr, Sikander Hayat, Rafael Kramann, Eva Kostenis, Jürgen Scheller, Jürgen Schrader

**Affiliations:** 1 Department of Molecular Cardiology,; 2 Biologisch-Medizinisches-Forschungszentrum (BMFZ), Genomics and Transcriptomics Laboratory, and; 3 Institute of Biochemistry and Molecular Biology II, Heinrich-Heine University, Düsseldorf, Germany.; 4 Department of Pharmaceutical Biology, University of Bonn, Bonn, Germany.

**Keywords:** Cardiology, Inflammation, Cytokines

## Abstract

Plasma IL-6 is elevated after myocardial infarction (MI) and is associated with increased morbidity and mortality. Which cardiac cell type preferentially contributes to IL-6 expression and how its production is regulated are largely unknown. Here, we studied the cellular source and purinergic regulation of IL-6 formation in a murine MI model. We found that IL-6, measured in various cell types in post-MI hearts at the protein level and by quantitative PCR and RNAscope, was preferentially formed by cardiac fibroblasts (CFs). Single-cell RNA-Seq (scRNA-Seq) in infarcted mouse and human hearts confirmed this finding. We found that adenosine stimulated fibroblast IL-6 formation via the adenosine receptor A2bR in a Gq-dependent manner. CFs highly expressed Adora2b and rapidly degraded extracellular ATP to AMP but lacked CD73. In mice and humans, scRNA-Seq revealed that Adora2B was also mainly expressed by fibroblasts. We assessed global IL-6 production in isolated hearts from mice lacking CD73 on T cells (CD4-CD73^–/–^), a condition known to be associated with adverse cardiac remodeling. The ischemia-induced release of IL-6 was strongly attenuated in CD4-CD73^–/–^ mice, suggesting adenosine-mediated modulation. Together, these findings demonstrate that post-MI IL-6 was mainly derived from activated CFs and was controlled by T cell–derived adenosine. We show that purinergic metabolic cooperation between CFs and T cells is a mechanism that modulates IL-6 formation by the heart and has therapeutic potential.

## Introduction

IL-6 is a pleiotropic cytokine that becomes elevated in the acute response to injury (1). After myocardial infarction (MI), serum levels of IL-6 increase rapidly, correlating with the severity of tissue injury, and remain elevated for several weeks (2). IL-6 mediates its biological roles through a hexameric complex composed of IL-6, its receptor IL-6R, and glycoprotein 130 (IL-6/IL-6R/gp130) (3). This complex, in turn, activates classical and *trans*-signaling mechanisms via the membrane-bound IL-6R and the soluble (s) IL-6R, respectively, to execute various biochemical functions (4).

In human studies, a reduction of IL-6 below the level of 1.65 ng/L by treatment with the IL-1β–targeting monoclonal antibody canakinumab reduced adverse cardiac events such as MI, stroke, and cardiovascular death in patients with atherosclerosis (5), and inhibition of the IL-6R by tocilizumab improved myocardial salvage in a recent study of patients with ST-elevation MI (STEMI) (6). However, there are also experimental studies reporting cardioprotection during short-term IL-6 signaling by inducing an antiapoptotic program that enhances cardiomyocyte survival but can result in cardiomyocyte hypertrophy in the long term (4). Little information is available about the cell types within the heart that preferentially produce IL-6 or the mechanisms by which ischemia regulates IL-6 formation. IL-6 is generally assumed to be formed by cardiomyocytes during ischemia and reperfusion, however, endothelial cells (ECs), cardiac fibroblasts (CFs), and immune cells (ICs) may also participate (7–9).

Ischemia is well known to release substantial amounts of ATP from various cell types, and this ATP is subsequently broken down by a cascade of ectonucleotidases into adenosine that can signal via 4 adenosine receptors (A1R, A2aR, A2bR, A3R) (10). The reported affinities for adenosine differ greatly between the receptor subtypes: the EC_50_ values for the A1R, A3R, A2aR, and A2bR are 0.31, 0.29, 0.7, and 29 μM, respectively (11). We have recently shown that under in vivo conditions, the A2bR indeed becomes activated after MI (12), and this was associated with enhanced IL-6 expression. It also is known that A2bRs physically interact with A2aRs, forming stable A2a-A2b heteromers (13) with a distinct pharmacology and new signaling properties. The biological in vivo implications of this finding, however, are not fully understood.

We have reported that adenosine formed by CD73 (AMP → adenosine) on T cells orchestrates, most likely by autocrine and paracrine effects on the A2aR and A2bR, the cardiac healing process after MI in the remodeling phase (14). Tissue hypoxia as it occurs during tissue ischemia is associated with HIF-dependent induction of the A2bR (15) that was also found to be upregulated in injured cardiomyocytes and ICs of the infarcted heart (14). Furthermore, the expression of IL-6 was reported to be substantially reduced in cardiomyocytes and ICs (not T cells) in A2bR-KO mice (12). These and additional findings suggested the presence of a targetable adenosine/A2bR/IL-6 axis, which is triggered by adenosine formed by the ischemic heart (12).

The primary site of A2bR expression in mice was reported to be the vasculature, particularly smooth muscle cells, ECs and macrophages, suggesting a potential role in angiogenesis (16). We recently reported that the secretion of IL-6 by epicardial stromal cells (EpiSCs) formed after MI is regulated by adenosine signaling via the A2bR, which is also highly expressed in this cell type (17). The A2bR is coupled to Gs and Gq proteins (18) and, in contrast to the other adenosine receptors, requires micromolar adenosine concentrations to become activated (19). Under conditions of hypoxia, transcription of the A2bR is upregulated, and extracellular adenosine is elevated to levels sufficient for A2bR activation (20).

In the present study, we defined the cardiac cell types involved in the post-MI formation of IL-6 and the role of purinergic signaling in this process. We report that CFs were the main source of IL-6 in the ischemic heart of mice and humans and show that A2bR stimulation increased IL-6 expression and secretion in CFs in a Gq-dependent fashion. There is most likely thus-far unappreciated metabolic cooperation between CFs and T cells, in that CF-derived AMP serves as a substrate for CD73 on T cells so that local adenosine becomes augmented and feeds back to modulate IL-6 formation in CFs.

## Results

### CFs show the highest expression of IL-6 in the post-MI heart and coexpress the A2bR.

**Figure 1:**
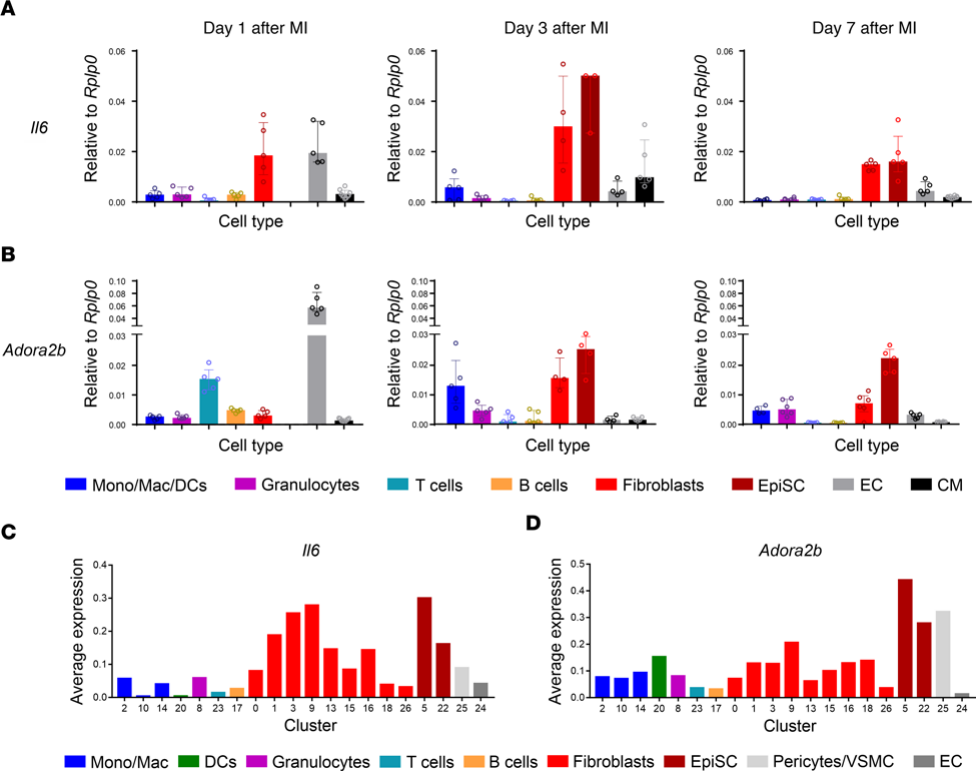
Temporal changes in expression profiles of *Il6* and *Adora2b* in different subpopulations of cardiac cells in the infarcted heart. (**A**) qPCR measurement of *Il6* mRNA expression in macrophages, monocytes, DCs, granulocytes, T cells, B cells, CFs, EpiSCs, ECs, and cardiomyocytes isolated from C57BL/6J mice on post-MI days 1, 3, and 7 (*n* = 3–5). (**B**) *Adora2b* mRNA expression in different cardiac cell populations (same post-MI time points and cells as in **A**) (*n* = 5). Values are the median with the IQR. (**C** and **D**) Analysis of *Il6* (**C**) and *Adora2b* (**D**) expression following scRNA-Seq analysis of hearts 5 days after MI. Populations of activated CFs, EpiSCs, and ICs isolated from the infarcted hearts of 3 C57BL/6J mice per group were combined (Supplemental Figure 1) and analyzed for their fractional contribution within the combined cluster (Supplemental Figure 3), resulting in 26 well-defined cell populations with the indicated cell identities. Mono, monocytes; Mac, macrophages; VSMC, vascular smooth muscle cells.

Numerous cell types were reported to secrete IL-6 within the heart (7-9). To explore the cell types preferentially involved in post-MI IL-6 secretion, we measured *Il6* transcripts in various cell types of the heart, such as CFs, epicardium-derived cells (EpiSCs), ECs, ICs, and cardiomyocytes that were isolated from the infarcted heart (21). To cover the early and late inflammatory response, analysis was done on post-MI days 1, 3, and 7. As shown in Figure 1A, the highest expression levels of *Il6* transcripts were detected on days 3 and 7 in CFs and EpiSCs. Clearly, the expression levels of *Il6* in various IC subtypes, and particularly cardiomyocytes, were generally lower. Since we previously reported that adenosine A2bR–dependent signaling enhances *Il6* expression in the ischemic heart (12), we further explored the cellular distribution of *Adora2b* expression. As with *Il6*, the highest expression levels of *Adora2b* were detected in CFs and EpiSCs that were closely related to CFs (22) (Figure 1B). Interestingly, *Adora2b*, one day after infarction, was highly expressed in ECs, which may have been related to the vascular A2bR signaling proposed as a central control point of hypoxia-associated vascular leakage (23).

Comparison of expression patterns of different cell types by quantitative PCR (qPCR) bears the risk of inaccuracies due to possible differences in housekeeping gene expression between 2 cell types. To this end, we have complemented a data set recently published by us on post-MI stromal cells (activated CFs [aCFs] and EpiSCs) (22) by an additional analysis of cardiac ICs that were isolated from the same infarcted hearts. Reanalysis revealed 26 genetically defined cell clusters (Supplemental Figure 1; supplemental material available online with this article; https://doi.org/10.1172/JCI163799DS1). Heatmap analysis (Supplemental Figure 2), combined with stringent analysis of the fractional contribution of aCFs, EpiSCs, and ICs within each of the combined clusters (Supplemental Figure 3), resulted in 19 genetically defined cell clusters.

At the single-cell level, we confirmed that cardiac stromal cells had the highest expression of *Il6* and, furthermore, that *Il6* was heterogeneously distributed among the different CF populations (Figure 1C). Interestingly, we observed strong *Il6* expression in fibroblast clusters 1, 3, and 9 that also showed elevated levels of either the activation marker periostin (*Postn*) or the myofibroblast marker *Acta2* (αSMA). Cluster 9, which showed the highest *Il6* expression levels, was characterized by the expression of several genes associated with the cell cycle and proliferation (*Cenpa*, *Stmn1*, *Ccnbc*, *Ube2c*, *Hist1h2ap*, *Top2a*, *Mki67)* and myofibroblast differentiation (*Acta2*, *Tpm2*, *Spp1*, *Timp1*), whereas clusters 1 and 3 clearly showed a myofibroblast signature (*Cthrc1*, *Fn1*, *Sfrp2*, *Ptn*, *Ddah1*), as was reported by several groups (22, 24, 25). Also, *Adora2b* (A2bR) expression by CFs and EpiSCs could be confirmed in the single-cell data (Figure 1D). Together, our data are consistent with the view that there may be a functionally relevant link between the A2bR and IL-6 formation in CFs of the post-MI heart. Exploration of additional genes that might be of interest can be done via the link to the combined scRNA-Seq data set using the user-friendly Shiny Cell tool (26) (https://visualisierung.gtl.hhu.de/data/52Publication/).

**Figure 2:**
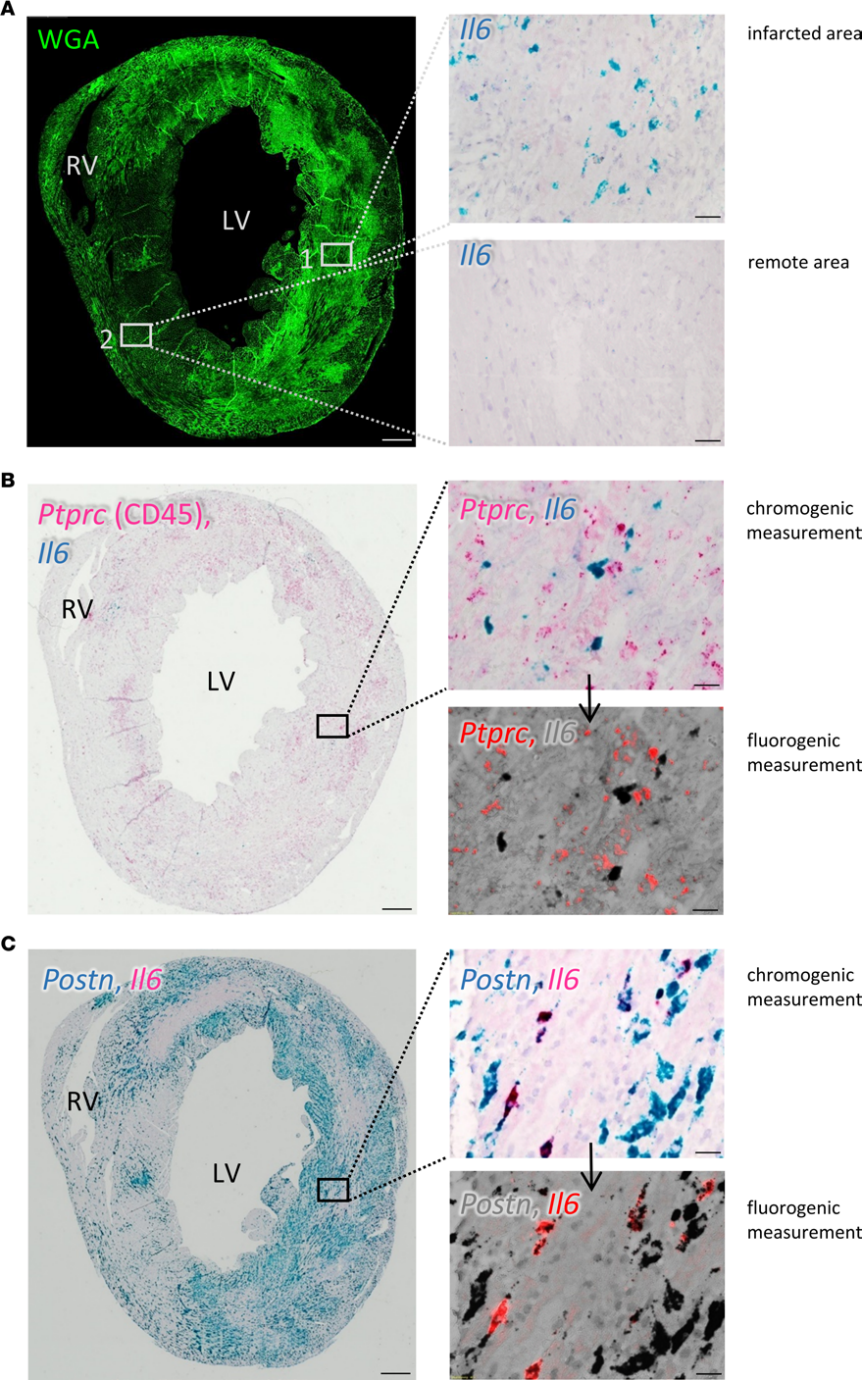
*Il6* expression in the infarcted heart as measured by RNAscope. Sections from hearts of C57BL/6J mice were analyzed 3 days after MI. (**A**) Representative WGA staining (bright green) delineates the infarcted area. Close-ups on the right were stained for *Il6* mRNA (blue) with RNAscope without counterstaining. (**B**) Representative section stained for *Ptprc* (CD45) (red) and *Il6* (blue) mRNA. Close-up in the upper right panel shows chromogenic staining at higher magnification. The chromogenic Fast Red dye could additionally be visualized in the red fluorescence spectrum, as shown in the lower panel of the close-up. The fluorescence image was overlaid with the bight-field image (gray). (**C**) Representative staining for *Postn* (blue) and *Il6* (red) mRNA. The upper and lower close-ups are the chromogenic and fluorescence images, respectively. Representative images of the same heart are shown. *n* = 3. Scale bars: 300 μm and 20 μm (close-up insets). LV, left ventricle; RV, right ventricle.

To further characterize the spatial distribution of *Il6* expression in the post-MI heart, we performed in situ hybridization and combined *Il6* with *Ptprc* (CD45) and *Postn* to mark ICs and activated fibroblasts, respectively. Fluorescence-labeled wheat germ agglutinin (WGA) (27), used for orientation, brightly stained the infarcted area (Figure 2A). Representative images in Figure 2A (infarcted area, inset 1) and Figure 2A (remote area, inset 2) show that the distribution of *Il6*-expressing cells was limited to the infarcted heart. Labeled cells were small and dispersed throughout the infarcted area (Figure 2A). The expression of *Il6* by RNAscope was not detectable in cardiomyocytes. The majority of cells expressing *Ptprc* (CD45) (red), as shown in representative tissue section images in Figure 2B, were negative for *Il6* (blue). Interestingly, some *Il6*-expressing cells were located in close proximity to ICs (Figure 2B, upper right panel). However, when *Il6* (red) and *Postn* (blue) were costained, the vast majority of *Il6*-expressing cells were also positive for *Postn* expression (Figure 2C). Note that a large fraction of *Postn*-expressing cells were negative for *Il6* expression. Since chromogenic staining in colocalization experiments may be difficult to interpret, particularly when one staining is much weaker than the other, we additionally performed RNAscope experiments with fluorophore-coupled probes and confirmed *Postn* and *Il6* colocalization (Supplemental Figure 4). Together, these findings again demonstrated that CFs were the major local producers of IL-6 in the infarcted heart.

**Figure 3:**
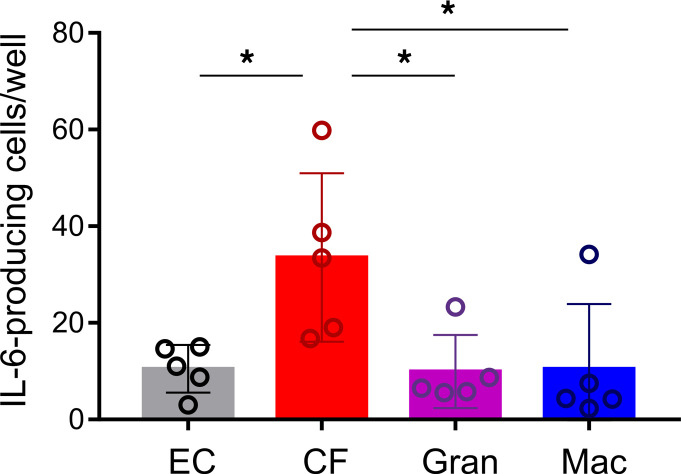
Quantification of IL-6 secretion from cardiac ECs, granulocytes, macrophages, and CFs by ELISPOT. Cells were isolated 3 days after MI and seeded at a density of 10,000 cells per well. The number of IL-6–secreting cells was determined by ELISPOT assay after overnight incubation. *n* = 5. Values are the mean ± SD. *P ≤ 0.05, by 1-way ANOVA with Tukey’s multiple-comparison test. Gran, granulocytes.

To confirm the above findings on the protein level, we sorted ECs, CFs, granulocytes, and macrophages from hearts of C57BL/6J mice 3 days after MI and performed ELISPOT assays. As summarized in Figure 3, CFs had the highest number of IL-6–secreting cells as compared with the other cell types analyzed. Like the RNAscope data (Figure 2), the ELISPOT results showed that only a small fraction of cells within 1 population were secreting IL-6 in measurable amounts.

### A2bR signaling in CFs.

**Figure 4:**
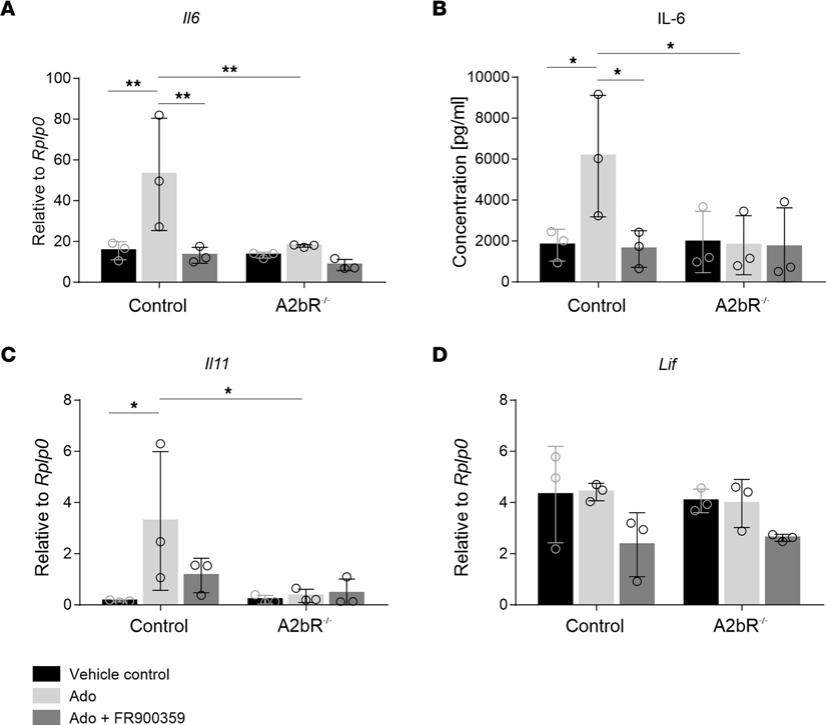
A2bR stimulation of CFs by adenosine induces IL-6 secretion in a Gq-dependent manner. (**A**–**D**) Murine CFs were isolated from A2bR^–/–^ or A2bR^+/+^ (control) transgenic mice and incubated in the presence or absence of 20 μM adenosine (Ado) or 20 μM adenosine in combination with the Gq inhibitor FR900359 (1 μM) in the presence of the adenosine deaminase inhibitor EHNA (33 μM) and the ENT1 inhibitor NBMPR (33 μM). (**A**, **C**, and **D**) *Il6*, *Il11*, and *Lif* expression was determined by qPCR 24 hours later. (**B**) IL-6 cytokine secretion was measured after 24 hours. *n* = 3. Values are the mean ± SD. *P ≤ 0.05 and **P ≤ 0.01, by 2-way ANOVA using the 2-stage step-up method of Benjamini, Krieger, and Yekutieli.

To further explore the link between adenosine, the A2bR, and IL-6, we treated cultured CFs with adenosine (20 μM) and measured the expression of *Il6* and *Il11*, both of which signal through a homodimer of the ubiquitously expressed β-receptor gp130, combined with a cytokine-specific receptor chain (28). As shown in Figure 4, A and B, adenosine increased IL-6 at both the mRNA and protein levels, and this effect was fully blocked by FR900359 (29), a specific blocker of the Gq signaling pathway. Likewise, the stimulatory effect of adenosine was absent in CFs isolated from A2bR-KO mice (Figure 4, A and B). Similar results were also obtained for *Il11* (Figure 4C). However, the IL-6 cytokine family member *Lif*, which was reported to compensate for the loss of IL-6 in a murine MI model (30), was not affected by adenosine (Figure 4D). Together, these findings show that adenosine in CFs signaled via the A2bR to induce IL-6 and *Il11* expression.

Aside from adenosine, pathogen- and danger-associated molecular patterns (PAMPs or DAMPs) are well known to strongly induce IL-6 expression by activation of a pattern recognition receptor (PRR) or indirectly via induction of IL-1β or TNF-α (28). To gain insight into the relative contribution of these factors, we analyzed the expression of *Il6*-inducing or -regulating genes using the single-cell data set described above. As summarized in Supplemental Figure 5B, the expression of IL-6 in cardiac stromal cells was in the same range as that of the IL-1β and TNF-α receptors Il1r1, Tnfrst1a, and Tnfrst1b. It is worth noting that the majority of transcripts known to be involved in *Il6* induction or regulation were clearly more abundant in cardiac ICs (Supplemental Figure 5B). This suggests that adenosine-mediated A2bR signaling, together with IL-1β and TNF-α, is likely to be a major player in IL-6 formation in activated CFs.

### Purinergic signaling in CFs.

**Figure 5:**
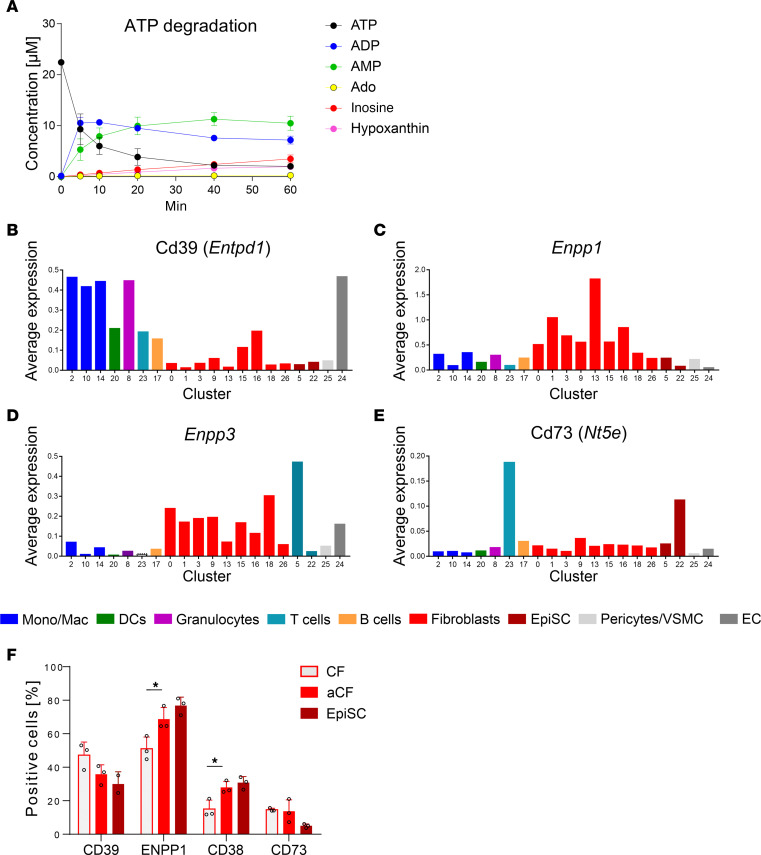
Purinergic signaling in CFs. (**A**) Kinetics of extracellular ATP degradation in murine CFs isolated from C57BL/6J mice analyzed by HPLC (reaction was started with 20 μM ATP, 37°C; *n* = 3). (**B**–**E**) Expression analysis of the ATP-degrading enzymes *Entpd1* (CD39) (**B**), *Enpp1* (**C**), *Enpp3* (**D**), and *Nt5e* (CD73) (**E**) by scRNA-Seq of 3 hearts 5 days after MI (see above and Supplemental Figures 1–3). (**F**) Protein expression analysis by flow cytometry of CD39, ENPP1, CD38, and CD73 in CFs obtained from noninfarcted hearts as compared with aCFs and EpiSCs from mice 5 days after MI (*n* = 5). Only ENPP1 was measured because specific antibodies against ENPP3 were not available. Values are the mean ± SD. *P ≤ 0.05, by 1-way ANOVA with Dunnett’s multiple-comparison test.

Since CFs can produce IL-6 by adenosine-mediated activation of the A2bR (Figure 4), we next explored whether CFs also have the ability to produce adenosine in significant quantities for autocrine signaling. To this end, we measured the kinetics of extracellular ATP degradation by HPLC. As shown in Figure 5A, CFs rapidly degraded ATP but failed to generate significant amounts of adenosine over a period of 40 minutes. Consequently, AMP accumulated over time, indicating negligible activity of the AMP-degrading enzyme CD73.

We further expanded this finding by analyzing various enzymes involved in adenosine formation using single-cell data. As shown in Figure 5B, CD39 (*Entpd1*) had the highest expression in macrophages and granulocytes and was only expressed at low levels in stromal cells. On the other hand, the pyrophosphatases *Enpp1* and *Enpp3* were mainly expressed in CFs, with only minor expression in cardiac ICs (Figure 5, C and D). The dominant role of ENPP1 and the low expression of CD73 were further confirmed at the protein level (Figure 5F). Consistent with data in the literature (14), we detected the highest expression of CD73 in T cells (EpiSC cluster 22 in Figure 5E). The ATP transporters pannexin 1 (*Panx*) and Cx43 (*Gja1*) were highly expressed in CFs (Supplemental Figure 7), whereas the vesicular nucleotide transporter Vnut (*Slc17a9*) showed the highest expression in T cells and B cells.

### CD73 expression on T cells modulates IL-6 secretion in post-MI hearts.

**Figure 6:**
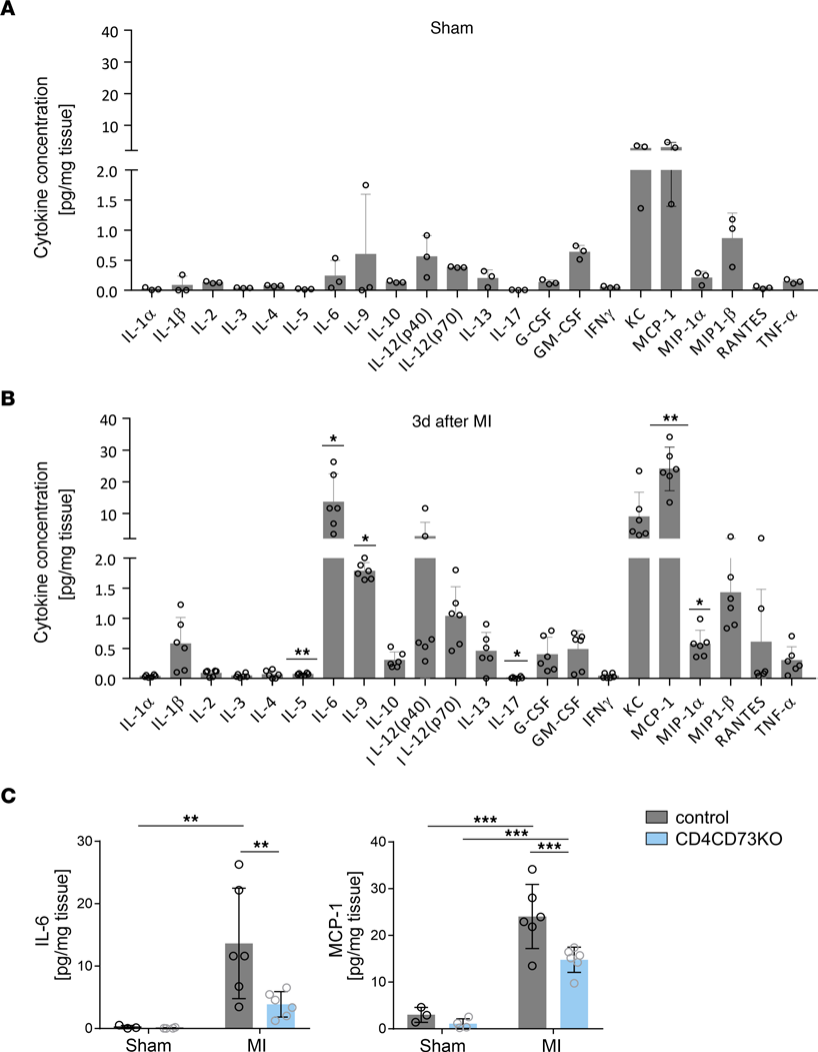
Cytokine secretion by the heart is influenced by MI and lack of CD73 on T cells. (**A**) Cytokine secretion was assessed in the coronary effluent of isolated perfused hearts from sham-operated CD4-Cre^–/–^ CD73^fl/fl^ mice (**A**) and 3 days after MI (**B**). Sham-operated mice, *n* = 3; MI mice, *n* = 6. (**C**) Influence of CD73 deficiency on T cells on the cardiac release of IL-6, MCP-1, and IL-9 measured in control (CD4-Cre^–/–^ CD73^fl/fl^) and T cell–specific CD73-KO (CD4-Cre^+/–^ CD73^fl/fl^) mice 3 days after MI (*n* = 6). Values are the mean ± SD. *P ≤ 0.05, **P ≤ 0.01, and ***P ≤ 0.001, by 2-tailed Student’s *t* test (**A** and **B**) and 2-way ANOVA using the 2-stage step-up method of Benjamini, Krieger, and Yekutieli (**C**).

To explore the functional relevance of adenosine in modulating cardiac IL-6 formation, which was suggested by our previous work (12, 17), we studied the infarcted heart. As a convenient readout for global cardiac cytokine formation, we assessed the release 23 cytokines in the coronary effluent perfusate of isolated perfused hearts that had previously been subjected to MI (50 minutes of ischemia/reperfusion, 3 days after MI). The coronary perfusion pressure was maintained at 80 mmHg. In these experiments, we used T cell–specific CD73-KO mice (14), in which we previously showed that the significant elevation of adenosine in the remodeling phase was almost fully blunted. Functionally, this metabolic condition was associated with no changes in infarct size but severe impairment during cardiac remodeling (14). As shown in Figure 6B, in vivo ischemia/reperfusion in WT mice as compared with sham-operated controls (Figure 6A) resulted in a significant increase in the release of IL-5, IL-6, IL-9, IL-17, monocyte chemoattractant protein 1 (MCP-1), and macrophage inflammatory protein 1α (MIP-1α) under comparable experimental conditions (14). We found that the expression of IL-6 and MCP-1 showed the greatest changes (IL-6 sham mean: 0.24 ± 0.25 pg/mL; IL-6 MI mean: 13.6 ± 8.8 pg/mL [57-fold]; MCP-1 sham mean: 3 ± 1.6 pg/mL; MCP-1 MI mean: 24 ± 6.8 pg/mL [8-fold], respectively). In mice lacking CD73 on T cells (CD4-Cre CD73^fl/fl^), the infarct-induced release of IL-6 and MCP-1 that is directly induced by IL-6 was significantly reduced (Figure 6C). These findings provide evidence, that adenosine formed by CD73 on T cells in the remodeling phase was quantitatively sufficient to significantly modulate cardiac IL-6 formation in the intact heart.

We next explored whether adenosine mainly produced by cardiomyocytes during an acute ischemic event and released into the coronary circulation immediately after the release of ischemia can alter the initial production of IL-6 by the heart. To this end, and to avoid the participation of T cells, we performed experiments in isolated saline-perfused mouse hearts subjected ex vivo to 30 minutes of ischemia followed by reperfusion. Similar to clinical studies (2), we found that release of ischemia was associated with a substantial liberation of IL-6 into the effluent perfusate in all experiments (Supplemental Figure 10). However, there were no significant changes in IL-6 release in the A2bR-KO heart. In the same experimental setting, we also measured adenosine and adenine nucleotides in the coronary effluent by sensitive HPLC methods. Data summarized in Supplemental Table 1 show that adenosine concentrations massively increased (17-fold) immediately after the release of ischemia, reaching a concentration of 5.2 μM. It is worth noting that immediately after the release of ischemia, the concentration of ATP reached a mid-micromolar concentration (11.3 μM, which amounts to a 50-fold increase above the normoxic baseline concentration). Together, these experiments show that the initial IL-6 formation immediately after release of ischemia was not under the control of the A2bR, despite the highly elevated extracellular adenosine levels.

## Discussion

IL-6, an inflammatory cytokine with pleiotropic effects in diverse organs, has been linked to adverse cardiovascular outcomes including MI (5, 6, 31, 32). The cellular source of post-MI IL-6, however, is not well defined and is reported to involve cardiomyocytes, fibroblasts, and ECs (7–9, 33). Interestingly, unstimulated myocytes have been reported to lack significant expression of *IL6* mRNA but do express significant levels when pharmacologically stimulated in vitro (7). Therefore, cardiomyocytes have the potential to produce IL-6, which, under the in vivo conditions used in the present study, was very minimal. Here, we demonstrate that IL-6 was mainly generated by activated fibroblasts in the post-MI heart and was modulated by CD73-derived adenosine produced by T cells. Furthermore, fibroblasts and T cells were likely to metabolically cooperate via fibroblast-derived AMP, which served as a substrate for T cell CD73.

The conclusion that *Il6* in the post-MI heart was predominantly expressed by CFs within the injured heart was supported by several lines of evidence: expression analysis of isolated cardiomyocytes and various noncardiomyocytes (Figure 1, A and B), single-cell sequencing (Figure 1C), RNAscope data (Figure 2), and measurements of IL-6 at the protein level (Figure 3). Our finding does not appear to be species specific. We observed that IL-6 was also strongly expressed in fibroblasts isolated from the infarcted human heart and showed only negligible expression in cardiomyocytes (Supplemental Figure 8). To address the question of how the IL-6 clusters evolve after infarction, we evaluated the human data set in more detail (34) and differentiated between healthy tissue (myogenic), acutely infarcted tissue (ischemic), and chronically fibrotic tissue (fibrotic). As shown in Supplemental Figure 9, IL-6 in ischemic tissue was clearly upregulated in fibroblasts and proliferating cells comprising mainly fibroblasts. In fibrotic tissue, IL-6 was predominantly expressed in proliferating cells. IL-6–expressing cell clusters also expressed Adora2B. Again, expression of Adora2B was low in human cardiomyocytes (Supplemental Figure 8). Similar to the mouse data, expression of Adora2B was high in pericytes. Together, these data suggest that in the human system as well, CFs are an important source of cardiac IL-6 that may be controlled by adenosine via the A2bR.

CFs are a major cell population within the heart that comprise approximately 20% of all noncardiomyocytes (35), and this number increases during scar formation. CFs are strategically well positioned within the injured heart so that IL-6 and other fibroblast-derived metabolites can readily reach surrounding target cells such as ICs and cardiomyocytes by diffusion. scRNA-Seq identified extensive bidirectional signaling of IL-6 family members between CFs and cardiac ICs: the receptor for IL-6 (IL-6R) was most prominently expressed on monocytes/macrophages and DCs in the post-MI heart. This finding complements the observation that IL-6, via classical IL-6 signaling, plays a role in monocyte differentiation into macrophages, thereby constituting an essential factor in the molecular control of antigen-presenting cell development (36). On the other hand, CFs strongly express *Il6st* (gp130), suggesting a role for IL-6 *trans*-signaling (Supplemental Figure 7). Notably, tocilizumab blocks both pathways (6) and thus cannot differentiate between classic and *trans*-signaling in the post-MI heart. Recently a novel approach was reported that exclusively blocks *trans*-signaling (mediated by sgp130Fc) compared with the use of anti–IL-6 antibodies (panantagonism) (37). The authors found that only sgp130Fc, but not anti–IL-6 antibodies, reduced infarct size and preserved ejection fraction (EF) in the remodeling phase. These data demonstrate that IL-6 predominantly acts via *trans*-signaling, inducing proinflammatory activity, which is involved in the deterioration of cardiac function. Since the extent of the ischemic injury (50 min/reperfusion), as well as the changes in IL-6 formation, was identical to that observed in the present study, a direct comparison of the data sets is possible. In functional terms, this means that adenosine derived from CD73 on T cells in the post-ischemic phase involves not only antiinflammatory activity (via the A2aR) but also the formation of proinflammatory activity (via A2bR-mediated IL-6). It is most likely the balance between these opposing functionalities that critically determines outcomes.

The functional role of IL-6 in the post-ischemic heart is critically dependent on the time after myocardial injury. In the acute phase, IL-6 activates ICs, directs IC trafficking, and reduces cardiomyocyte apoptosis (4). Chronically elevated IL-6 levels, on the other hand, lead to chronic inflammation, enhanced tissue fibrosis, and cardiomyocyte hypertrophy (4). Recently, in the randomized, double-blind, placebo-controlled clinical trial ASSAIL-MI (ASSessing the Effect of Anti-IL-6 Treatment in Myocardial Infarction), IL-6R inhibition with tocilizumab, a humanized anti–IL-6R monoclonal antibody, significantly attenuated inflammation and increased myocardial salvage in STEMI patients (6). In mice, however, blockade of the IL-6R by a monoclonal antibody did not alter the process of cardiac remodeling (38). Similarly, a lack of IL-6 in KO mice did not affect long-term MI size or left ventricular function, remodeling, or survival (30). KO of IL-6 resulted in marked STAT3 phosphorylation, along with strong induction of angiotensin II in the infarcted left ventricle. This went along with induction of the angiotensin II receptor type 1 (AT1R) and the IL-6 family member leukemia inhibitory factor (LIF), which also signal via JAK/STAT in IL-6–deficient mice. This strongly suggests, the authors argue (30), that compensatory mechanisms are activated by the chronic loss of IL-6. Most likely, the transgenic model did not mimic the acute loss of IL-6. In fact, when post-MI IL-6 is reduced acutely by administration of the IL-6R inhibitor tocilizumab, it enhanced myocardial salvage in patients with acute STEMI (6). It is also worth noting that tocilizumab induced a rapid reduction in neutrophils in patients with STEMI, which may be related to its beneficial effects on myocardial salvage (39).

Little is known about the link between tissue ischemia and IL-6 and endogenous factors that modulate IL-6 production in vivo. IL-6 increases in response to TLR agonists, cytokines (e.g., TNF-α and IL-1β), and lipid mediators (e.g., prostaglandins) and as a consequence of cellular stress (40). Fibroblast IL-6 formation was recently reported to be under the control of the atypical chemokine receptor 4 (ACKR4), the overexpression of which aggravated post-MI functional impairment (41). Analysis of single-cell data suggests that, aside from the A2bR, signaling of IL-1β and TNF-α is important in the production of IL-6 by activated CFs (Supplemental Figure 5B).

Tissue hypoxia associated with MI is well known to be a potent stimulus for the production of adenosine (42), which exerts powerful A2aR-mediated antiinflammatory activity (42, 43) and enhances cardiac IL-6 formation (12). We have previously reported that CD73 (found to be most highly expressed on T cells in the present study; Figure 5E) promotes cardiac healing following MI (14). Furthermore, using MRI, we previously extensively characterized the functional outcome over time in mice when CD73 was deleted either globally (44) or on T cells (14). In all cases, we determined the infarct size by gadolinium MRI and found that in both genetic models, the functional outcomes were identical: no influence on infarct size but a severe reduction in EF and a significantly attenuated and prolonged healing process (14). In the latter study, we also showed that the release of adenosine into the coronary effluent perfusate was significantly elevated in the post-ischemic phase and that loss of CD73 almost fully abolished coronary adenosine release (Figure 3 in ref. 44). Since the genetic model and extent of ischemia/reperfusion in the present study and our previous findings were identical, the data sets can be directly compared. This means that the marked reduction of IL-6 secretion in mice lacking T cell–derived adenosine was associated with adverse cardiac remodeling. Given these new findings, the original pathophysiological concept (14) must be expanded: not only does T cell–derived adenosine feed back to T cells in an autocrine fashion and have paracrine activity, it also exerts endogenous control over post-ischemic IL-6 formation.

Signaling of adenosine-induced IL-6 release in CFs was fully mediated by the A2bR involving Gq (Figure 4). We additionally found that both CFs and EpiSCs highly expressed the A2bR on post-MI days 3 and 7 (Figure 1). Also, in the infarcted human heart, CFs showed robust ADORA2B expression (Supplemental Figure 8). The similar cellular distribution of *Adora2b* and *Il6* supports the proposed signaling axis: CD73-derived adenosine → Adora2b receptor activation → IL-6 formation by CFs. Interestingly, pharmacological activation of the A2bR has been reported to reduce myocardial reperfusion injury by promoting antiinflammatory macrophage differentiation (45). The extent to which the observed cardioprotection involves IL-6 production by cardiac stromal cells remains to be elucidated.

While adenosine via the A2bR was an important regulator of cardiac IL-6 formation in the remodeling phase, it did not appear to influence IL-6 immediately after release of acute ischemia (Supplemental Figure 10). The most likely explanation for this finding is that expression of the A2bR in the unstressed heart was very low and that upregulation — known to be transcriptionally induced by HIF — required 18–24 hours under in vitro and in vivo conditions, as previously reported (23, 46). Interestingly, we observed strong release of ATP immediately after the release of ischemia, reaching mid-micromolar concentrations in the extracellular space (Supplemental Table 1), which is known to stimulate IL-6 formation (47). Whether ATP modulates initial IL-6 formation immediately after ischemia remains to be explored in future studies.

Adenosine formed by the ischemic heart has multiple functions (48); it strongly inhibits tissue inflammation by activation of A2aRs on ICs (49), stimulates the formation of IL-6 by CFs via the A2bR, and triggers, via Adora2B, normoxic HIF-1α induction in the CFs and EpiSCs formed after MI (50). Whether the A2bR and HIF-1α synergize to protect the injured heart remains to be elucidated.

We have previously reported that adenosine formed by T cell CD73 in vivo is quantitatively sufficient to induce wound healing after MI (14). In view of the small number of CD73-positive T cells in the infarcted myocardium, the observed substantial functional changes were rather surprising. This discrepancy can be explained by our finding that AMP, the substrate for T cell CD73, was not solely formed by activated T cells but was derived from surrounding cells as well. Since CFs are largely devoid of CD73, but readily form AMP from extracellular ATP (Figure 5A), it is plausible that AMP is supplied by CFs and reaches T cells by diffusion, thereby enabling efficient production of adenosine. The present study confirms that, among the noncardiomyocytes of the infarcted heart, the expression of CD73 was by far the highest on T cells (Figure 5E). Similar to CFs, granulocytes and monocytes/macrophages also showed high expression of CD39 (*Entpd1*) but only little CD73 (*Nt5e*) (Figure 5, B and E), again pointing to the accumulation of AMP. Finally, it is possible that extracellular AMP within the intact heart may be derived from sympathetic nerve terminals (51). In summary, these findings point to an important trafficking of purines within the interstitial space that metabolically links AMP production by various cell types to T cell CD73.

**Figure 7:**
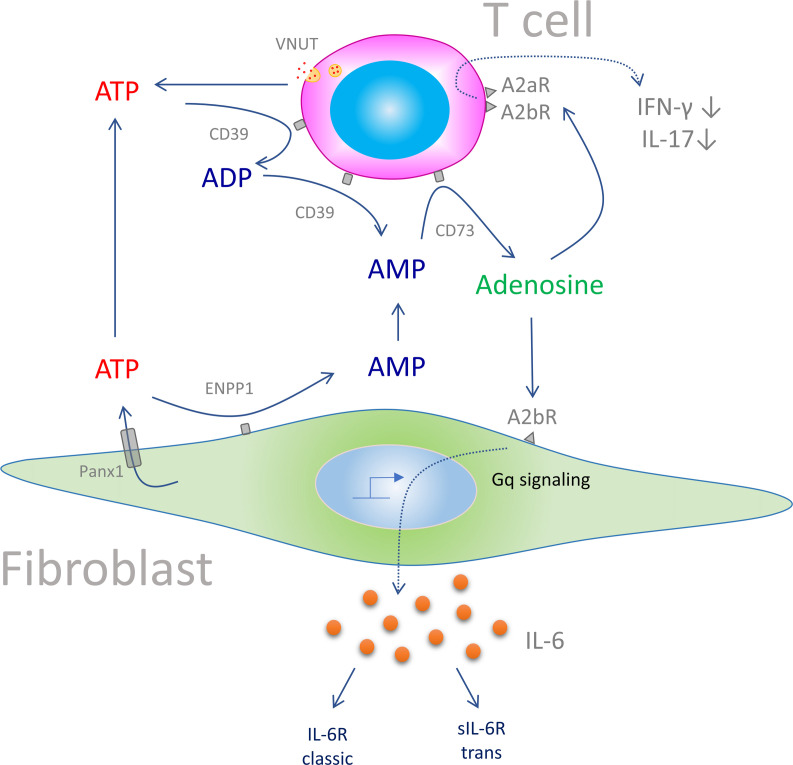
Scheme of the proposed mechanism by which purinergic crosstalk between T cells and CFs controls IL-6 production. In the post-MI heart during scar formation, ATP is derived mainly from noncardiomyocytes. Fibroblasts, in contrast to T cells, cannot further degrade AMP to adenosine, such that accumulating AMP diffuses to neighboring T cells, which highly express CD73. Similarly, granulocytes and monocytes contribute to this extracellular AMP pool. Supply of AMP to T cell CD73 augments local adenosine formation, which stimulates IL-6 production by fibroblasts via the A2bR in a Gq-dependent manner. T cells are not only the hub for extracellular adenosine formation, as adenosine can also modulate in an autacoid feedback loop the production of INF-γ and IL-17 (14). IL-6 predominantly acts via *trans*-signaling (sIL-6R) to confer proinflammatory activity in the post-MI heart (37).

The proposed metabolic link between T cells and CFs within the infarcted heart is schematically outlined in Figure 7. According to this view, CD73 on T cells is the catalytic hub that integrates the degradation of interstitial AMP derived from various cell types, particularly CFs, to produce significant quantities of adenosine. T cell–derived adenosine then feeds back to CFs to induce the formation of IL-6 by activating the A2bR in a Gq-dependent manner. At the same time, T cell–derived adenosine decreases by autocrine action the secretion of the proinflammatory and profibrotic cytokines INF-γ and IL-17 (14). Reported scRNA-Seq data also offer some insights into the cellular distribution of ATP-degrading enzymes. While ATP breakdown mainly depended on CD39 in granulocytes and monocytes/macrophages (Figure 5B), CFs predominantly expressed the pyrophosphatases ENPP1/-3 (Figure 5, C and D). Among the genes associated with cellular ATP release, CFs mainly expressed *Panx1*, whereas on T cells, Vnut (*Slc17a9*) expression dominated (Supplemental Figure 6).

CFs are increasingly recognized to play an important role in cardiac injury and repair (52). This involves the dynamic interaction of CFs and surrounding ICs but is also likely to occur at the cytokine level. Our scRNA-Seq data show that *Il6r* and *Lif* are mainly expressed by ICs, suggesting signaling from CFs to ICs (Supplemental Figure 7). Conversely, CFs are the likely target of oncostatin M (OSM), another member of the IL-6 family), which is mainly produced by ICs (Supplemental Figure 7). This fits with the observation that OSM attenuates post-MI remodeling and dysfunction (53) and promotes cardiac regeneration (54). There is also evidence suggesting that paracrine fibroblast-to-myocyte signaling involving IL-6 secretion regulates cardiomyocyte hypertrophy (55).

Given the important role of IL-6 and interconnected adenosine signaling pathways in cardiac injury and healing, some new therapeutic options can be considered. A first option is to augment endogenous adenosine formation, e.g., by inhibition of adenosine kinase, which phosphorylates adenosine back to AMP. Inhibition of this enzyme was recently reported to reduce infarct size, improve cardiac function, and prevent cell apoptosis and necroptosis in a mouse model of MI (56). Strategies to target the hypoxia-adenosine link that could be considered for a clinical trial were recently competently reviewed (57). Second, pharmacological blockade of the A2bR should reduce IL-6 production, which in turn is expected to be beneficial for cardiac healing. In line with this, systemic application of an A2bR antagonist (CS-62019) strongly attenuated cardiac remodeling after acute MI in the mouse (58) and rat (59). So far only a few A2bAR antagonists have reached clinical trials (60). Third, it might be sensible to combine the blockade of *trans*-signaling of IL-6, e.g., with sgp130Fc (37), with the augmentation of endogenous adenosine formation. Such a combination therapy should potentiate the cardioprotective effects of IL-6 *trans*-signaling blockade.

In summary, our study demonstrates that IL-6 in the post-MI heart was preferentially formed by CFs and that purinergic crosstalk between T cells and CFs via AMP is likely important for producing significant quantities of adenosine. This regulatory circle could be exploited in the future to promote cardiac healing after injury.

## Methods

### Animals.

Experimental animals were used at 8–12 weeks of age and had a body weight of 20–25 g. Male C57BL/6J mice (Janvier), T cell–specific CD73-KO mice (CD4-Cre^+/–^ CD73^fl/fl^), and control mice (CD4-Cre^–/–^ CD73^fl/fl^), also on a C57BL/6J background, as well asA2bR^–/–^ mice and their littermate controls on a C57BL/6N background (generated by Deltagene, RRID: MGI:3812454 and provided by Alexander Pfeifer, University of Bonn, Bonn, Germany), were housed and/or bred at the “Zentrale Einrichtung für Tierforschung und Tierschutzaufgaben” (ZETT) at Heinrich-Heine-University (Düsseldorf, Germany).

### Infarct model.

Reperfused MI was induced as previously described (61). In brief, 8- to 12-week-old male mice were anesthetized (isoflurane 1.5% via respiration), and the left anterior descending coronary artery was ligated for 50 minutes followed by reperfusion. Left anterior descending coronary artery occlusion was ensured by ST-segment elevation in ECG recordings. Mice were kept under analgesic treatment for 3 days (buprenorphin 0.1 mg/kg, 3 times/day). All animals used in this study survived the ischemia and reperfusion period.

### Quantification of gene expression by qPCR.

mRNA from cardiac cells was prepared using the RNeasy Micro Plus kit (QIAGEN) according to the manufacturer’s protocol. The quality and quantity of extracted mRNA were analyzed with the Agilent Bioanalyzer 2100 (Agilent Technologies). cDNA was generated using the High Capacity cDNA kit (Applied Biosystems). Because of the low amounts of cDNA, a preamplification reaction was performed using Pre-Amp Master Mix (Applied Biosystems), together with the same TaqMan assays (QIAGEN) later used for gene expression analysis in a reaction with 14 cycles. Gene expression was analyzed using Gene Expression Master Mix (Applied Biosystems) on a StepOnePlus Real-Time PCR system (Applied Biosystems). The following TaqMan primers were used: mouse *Rplp0* (housekeeping gene) (Mm00725448_s1), *Il6* (Mm00446190_m1), *Il11* (Mm00434162_m1), *Adora2b* (Mm00839292_m1), *Entpd1* (CD39) (Mm00515447_m1), *ENPP1* (Mm01193761_m1), *ENPP3* (Mm01193723_m1), *CD38* (Mm01220906_m1), and *Nt5e* (CD73) (Mm00501910_m1) (all from Applied Biosystems).

### In situ hybridization.

Formalin-fixed, paraffin-embedded hearts collected from mice 3 days after MI were stained with Alexa Fluor 488–conjugated WGA (Thermo Fisher Scientific) to visualize the infarct area. Additionally, sections from the same section plane were processed using the RNAscope 2.5 HD Duplex kit (Advanced Cell Diagnostics ADC) to visualize *Il6* (probe Mm-IL6 no. 315891 or probe Mm-IL6-C2 no. 315891-C2), CD45 (*Ptprc*) (probe Mm-Ptprc-C2 no. 318651), and *Postn* (probe Mm-Postn no. 418581) mRNA according to the manufacturer’s instructions. Sections were counterstained with Gill’s Hematoxylin I (Santa Cruz Biotechnology). Fluorescent images were taken from fixed (4% PFA in PBS) frozen section of hearts 3 days after MI and processed with the RNAscope Multiplex Fluorescent Reagent Kit (version 2) according to the manufacturer’s instructions. Images were recorded on an Olympus Bx 61 Microscope.

### Cardiac cell isolation and flow cytometry.

Cardiac cells were isolated by a previously described modified protocol (21). In brief, hearts were digested by intracoronary delivery of a collagenase solution (12,00 U/mL; collagenase type II from *Clostridium histolyticum*, Worthington) until the perfusion pressure was below 10 mmHg. To simultaneously isolate EpiSCs from the surface of infarcted hearts, hearts were additionally bathed in collagenase solution as described previously (21). Isolated single-cell suspensions were used for cardiac cell isolation by flow cytometric cell sorting or magnetic bead–mediated enrichment. For flow cytometric cell sorting, FcR binding sites were blocked (Miltenyi Biotec), and cells were stained for flow cytometric sorting on a MoFlo XDP flow cytometer (Beckman Coulter) with the following fluorochrome conjugated antibodies: CD45 Alexa Fluor 647 (30-F11, BD Biosciences); CD3 PE-CF594 (145-2C11, BD Biosciences); CD11b APC-Cy7 (M1/70, Thermo Fisher Scientific); Ly6G FITC (1A8, BD Biosciences); and CD19 PE (1D3, BD Biosciences). The cell populations analyzed were defined as follows: T cells, CD45^+^CD3^+^CD19^–^CD11b^–^; B cells, CD45^+^CD3^–^CD19^+^CD11b^–^; granulocytes, CD45^+^CD3^–^CD19^–^CD11b^+^Ly6G^+^; and monocytes, macrophages, and DCs, CD45^+^CD3^–^CD19^–^CD11b^+^Ly6g^–^. ECs, CFs, and EpiSCs were isolated using the following antibodies: CD31 APC (Mec 13.3, BD Biosciences) and CD45 PE-Cy7 (30-F11, BD Biosciences), whereas CFs and EpiSCs were defined as being CD45^–^CD31^–^ and ECs as CD45^–^CD31^+^. For the isolation of CFs by magnetic bead–mediated enrichment, a cardiac single-cell suspension was depleted of CD45^+^ and CD31^+^ cells by magnetic bead negative selection (Mojosort Nanobeads, BioLegend) according to the manufacturer’s instructions, with the addition of biotin-labeled anti-CD31 (clone MEC13.3, BioLegend) antibody.

### IL-6 ELISPOT assay.

ECs, granulocytes, macrophages, or CFs were isolated from the hearts of C57BL/6J mice 3 days after MI (see above) and were seeded (10,000 cells per well each) onto an ELISPOT membrane provided in a commercial ELISPOT Mouse/Rat IL-6 Kit (EL406, R&D Systems) according to the manufacturer’s instruction. Cells were cultivated overnight in IMDM containing 10% FCS, 1% penicillin and streptomycin, 1% GlutaMAX, and 25 μM β-mercaptoethanol. IL-6–positive spots were quantified using ImageJ software (NIH) (62). First, RGB images were split into hue, saturation, and brightness stacks; the image representing the brightness was used to set a threshold (Yen), and spots were counted using the “analyze particle” tool.

### IL-6 expression and secretion assay.

Murine CFs were isolated from healthy A2bR^–/–^ mice or their littermate controls by collagenase digestion using the Langendorff methods and magnetic bead–mediated enrichment as described above. Isolated CFs were cultivated in DMEM complete (4,500 mg/L glucose, 20% FCS, 1% penicillin and streptomycin, 1% GlutaMAX). All experiments were conducted with cells in passage 2: 60,000 CFs were seeded per well of a 12-well plate and permitted to settle overnight (passage 2). Cells were washed with DMEM complete and incubated with 400 μL DMEM complete containing 33 μM erythro-9-(2-hydroxy-3-nonyl) adenine (EHNA) (an adenosine deaminase [ADA] inhibitor, Tocris) and 33 μM nitrobenzylthioinosine (NBMPR) (an inhibitor of equilibrative nucleoside transporter 1 ENT1, Tocris), with or without 1 μM FR900359 (a Gq inhibitor, provided by Evi Kostenic, University of Bonn, Bonn, Germany) for 10 minutes at 37°C. Afterwards, 100 μL DMEM complete was added and contained (besides 33 μM EHNA and 33 μM NBMPR with or without 1 μM FR900359) 100 μM adenosine (Tocris) or only the corresponding amount of DMSO (vehicle control), giving a final concentration of 20 μM adenosine. After incubation for 24 hours, supernatant was collected and used for cytokine analysis with Bioplex technology (Bio-Rad) or IL-11 ELISA (Thermo Fisher Scientific). Cells were washed with PBS and used for mRNA expression analysis.

### ATP degradation assay.

CFs isolated as described above (magnetic bead isolation) from hearts of healthy C57BL/6J mice were seeded at a density of 10,000 cells per well of a 96-well, flat-bottomed plate (1 well per time point) and allowed to settle overnight. Cells were incubated with 20 μM ATP (MilliporeSigma) dissolved in HBSS buffer (Life Technologies, Thermo Fisher Scientific). Supernatants were collected at the indicated time points, and ATP metabolite concentrations were determined by HPLC (Waters) at 254 nm.

### Flow cytometric determination of ATP- und ADP-degrading enzymes.

For FACS analysis of ATP- and AMP-degrading enzymes on CFs (healthy mice), aCFs, and EpiSCs (day 5 after MI), hearts were digested as described above and stained with CD45 APC (30-F11, BD Biosciences), CD45 PE-Cy7 (30-F11, BD Biosciences), CD31 BV510 (MEC13.3, BD Biosciences), CD39 PeCy7 (24DM51, BD Biosciences), CD38 APC (90, BioLegend), CD73 FITC (496406, R&D Systems), ENPP1 APC (YE1/19.1, BioLegend), MEFSK-4 PE (mEF-SK4, Miltenyi Biotec), CD90.2 PE (30-H12, Thermo Fisher Scientific), or PDGFRA PE (ADA5, eBioscience). Dead cells were excluded by 7AAD staining. Cardiac stromal cells were identified by gating on 7AAD^–^CD45^–^CD31^–^ cells that were positive for the PE stain, which was a mixture of MEFSK-4, CD90.2, and PDGFRA.

### Single-cell transcriptomics.

For single-cell transcriptomics, hearts were digested as described above, followed by FACS for the following cell populations: CD31^–^CD45^+^ (ICs) and CD31^–^CD45^–^ (stromal cells), whose identity (CFs or EpiSCs) was defined by cell localization (see above) (21). The sorted single-cell suspensions were directly used for the scRNA-Seq experiments (as was already reported for aCF and EpiSC populations; ref. 22). Here, the analysis reported in Hesse et al. (22) was combined with our IC analysis of the same hearts studied by Hesse et al., revealing a new clustering for these analyses. scRNA-Seq analysis was performed by 10x Genomics Chromium System (10x Genomics). Cell viability and cell number analyses were performed via trypan blue staining in a Neubauer counting chamber. A total of 2,000–20,000 cells, depending on cell availability, were used as input for the single-cell droplet library generation on the 10x Chromium Controller system utilizing the Chromium Single Cell 3′ Reagent Kit, version 2, according to the manufacturer’s instructions. Sequencing was carried out on a HiSeq 3000 system (Illumina) according to manufacturer’s instructions, with a mean sequencing depth of approximately 90,000 reads/cell for EpiSCs and approximately 70,000 reads/cell for aCFs as well as approximately 55,000 reads/cell for ICs. Differences in sequencing depth were necessary in order to achieve a similar sequencing saturation of approximately 70% between all samples.

### Processing of scRNA-Seq data.

Raw sequencing data were processed using 10x Genomics CellRanger software (version 3.0.2). Raw BCL files were demultiplexed and processed to Fastq-files using the Cell-Ranger mkfastq pipeline. Alignment of reads to the mm10 genome and unique molecular identifier (UMI) counting were performed via the CellRanger count pipeline to generate a gene-barcode matrix. The median number of detected genes per cell was 3,155 for EpiSCs, 3,265 for aCFs, and 2,028 for ICs. The median of UMI counts per cell was 10,689 for EpiSCs, 11,110 for aCFs, and 6,096 for ICs. Mapping rates (reads mapped to the genome) were approximately 89% for EpiSCs, 90.9% for aCFs, and 88% for ICs.

### Filtering and clustering of scRNA-Seq data.

Further analyses were carried out with the Seurat version 3.0 R package (63). Initial quality control consisted of the removal of cells with fewer than 200 detected genes as well as the removal of genes expressed in fewer than 3 cells. Furthermore, cells with a disproportionately high mapping rate to the mitochondrial genome (mitochondrial read percentages >5.0 for EpiSCs and aCFs and >7.5 for ICs) were removed, as they represented dead or damaged cells. Normalization we performed utilizing SCTransform. Biological replicates (*n* = 3) were integrated into 1 data set by identifying pairwise anchors between data sets and using the anchors to harmonize the data sets. Dimensional reduction of the data set was achieved by principal component analysis (PCA) based on identified variable genes and subsequent uniform manifold approximation and projection (UMAP) embedding. The number of meaningful principal components (PCs) was selected by ranking them according to the percentage of variance explained by each PC, plotting them in an “Elbow Plot,” and manually determining the number of PCs that represented the majority of variance in the data set. Cells were clustered using the graph-based clustering approach implemented in Seurat, version 3.0. Doublet identification was achieved with the DoubletFinder (version 2.0.2) (64) tool to generate artificial doublets using the PC distance to find each cell’s proportion of artificial k nearest neighbors (pANNs) and ranking them according to the expected number of doublets. Heatmaps were generated using Morpheus (https://software.broad-institute.org/morpheus).

A list of the marker genes for all clusters is available in Supplemental Data File 1. The average expression values of the combined clusters are provided in Supplemental Data File 2, and the average expression values of each underlaying analysis are available in Supplemental Data File 3 (aCFs), Supplemental Data File 4 (EpiSCs), and Supplemental Data File 5 (ICs). An interactive Shiny-based visualization of the single-cell data set generated via ShinyCell (26) is available at: https://visualisierung.gtl.hhu.de/data/52Publication/

### Isolated perfused Langendorff heart.

Hearts were excised from heparinized T cell–specific CD73-KO (CD4-Cre^+/–^ CD73^fl/fl^) or control (CD4-Cre^–/–^ CD73^fl/fl^) mice immediately after cervical dislocation, prepared in ice-cold standard Krebs-Henseleit-Buffer, mounted to the Langendorff perfusion set-up, and perfused with oxygenated Krebs-Henseleit buffer (37°C) at a constant coronary perfusion pressure of 100 cm H_2_O. After an equilibration duration of 15 minutes, cardiac effluent was collected for 15 minutes on ice in a beaker containing a protease inhibitor cocktail (cOmplete Tablets, Mini, EDTA-free, EASYpack, Roche). Cardiac effluent perfusate was concentrated using an Amicon Ultra 3K Centrifugal Filter Device to a final volume of approximately 200 μL. Cytokine concentrations were determined using a Bio-Plex Pro Mouse Cytokine 23-plex Assay (Bio-Rad) and normalized to the heart weight.

### IL-6 and various purines released into the coronary effluent perfusate of isolated hearts.

IL-6, ATP, ADP, AMP, adenosine, and inosine were measured in the coronary effluent perfusate from isolated saline-perfused hearts (Langendorff), which were subjected to 30 minutes of ischemia at 37^o^C. For IL-6 measurement, perfusates were collected prior to a 20-minute ischemia period (normoxic controls) and 1–20 minutes and 21–40 minutes after release of ischemia in WT and A2bR^–/–^ mice. Perfusates were treated with proteinase inhibitor (Complete Ultra Tablets EDTA-free, Roche) and concentrated using 10 kDa centrifugation filters (Amicon). IL-6 was measured by BioPlex (Bio-Rad). For the measurement of purinergic metabolites, aliquots from coronary perfusate (100 μL) were taken (without proteinase inhibitor treatment) at baseline and 1, 10, and 30 minutes after ischemia. Purinergic metabolites were measured by HPLC directly from unconcentrated aliquots using an ACQUITY UPLC Bio H-Class System equipped with a Cortecs C18+ UPLC column (3.0 × 150 mm, particle size 1.6 μm) (Waters). Separation of purine compounds was performed using a liner gradient of buffer A (150 mM KH2PO4/150 mM KCl, pH 6) and buffer B (150 mM KH2PO4 150 mM KCl/7.5% acetonitrile, pH 6). Purine absorbance was detected at 254 nm with baseline correction (Absorbance-MBF) (WT, *n* = 5; A2bR^–/–^, *n* = 4).

### Statistics.

Data are presented as the mean ± SD with the exception of Figure 1, in which the values are shown as the median with the IQR. The number of biological replicates is indicated in the figure legends. In cases where technical replicate experiments were performed, the mean value were are indicated as 1 “*n*.” Data were analyzed using GraphPad Prism (GraphPad Software) with either a 2-tailed Student’s *t* test or 1- or 2-way ANOVA with the corresponding post test. A *P* value of less than 0.05 was considered statistically significant.

### Study approval.

Animal experiments were performed in accordance with the national guidelines for animal care and were approved by the Landesamt für Natur-, Umwelt- und Verbraucherschutz of the state of North Rhine Westphalia (LANUV NRW, Recklinghausen, Germany).

## Author contributions

CA, J Scheller, and J Schrader conceived and designed the research study. CA conducted most of the experiments, acquired and analyzed data, and prepared figures and tables. ZD, ASH, CO, and TL conducted experiments and acquired and analyzed data. EK provided reagents, and JB analyzed data and prepared figures. RK and SK conducted human scRNA-Seq data analysis. JH and J Scheller provided critical advice and discussions. CA and J Schrader wrote the manuscript.

## Supplementary Material

Supplemental data

Supplemental data set 1

Supplemental data set 2

Supplemental data set 3

Supplemental data set 4

Supplemental data set 5

## Version 1 03/21/2023

In-Press Preview

## Version 2 06/01/2023

Electronic publication
