# Supplementary material for: IL-6 in the infarcted heart is preferentially formed by fibroblasts and modulated by purinergic signaling
Source: J Clin Invest. 2023 Jun 1;133(11):e163799. doi: 10.1172/JCI163799 (PMC10232006; doi:10.1172/JCI163799)
Supplement: Supplemental data [file jci-133-163799-s112.pdf]

Supplementary Material

for

IL-6 in the infarcted heart is preferentially formed by fibroblasts and modulated by purinergic signaling

Christina Alter[1], Anne-Sophie Henseler[1], Christoph Owenier[1], Julia Hesse[1], Zhaoping Ding[1], Tobias Lautwein[2], Jasmin Bahr[1], Sikander Hayat[3], Rafael Kramann[3], Eva Kostenis[4], Jürgen Scheller[5], Jürgen Schrader[1]

[1]Department of Molecular Cardiology, [2]Biologisch-Medizinisches-Forschungszentrum (BMFZ), Genomics & Transcriptomics Laboratory and [5]Institute of Biochemistry and Molecular Biology II, Heinrich-Heine-University Düsseldorf, Germany, [4]Department of Pharmaceutical Biology, University of Bonn, Germany, [5]Institute of Experimental Medicine and Systems Biology, RWTH Aachen University, Germany.

Supplemental Figure S1

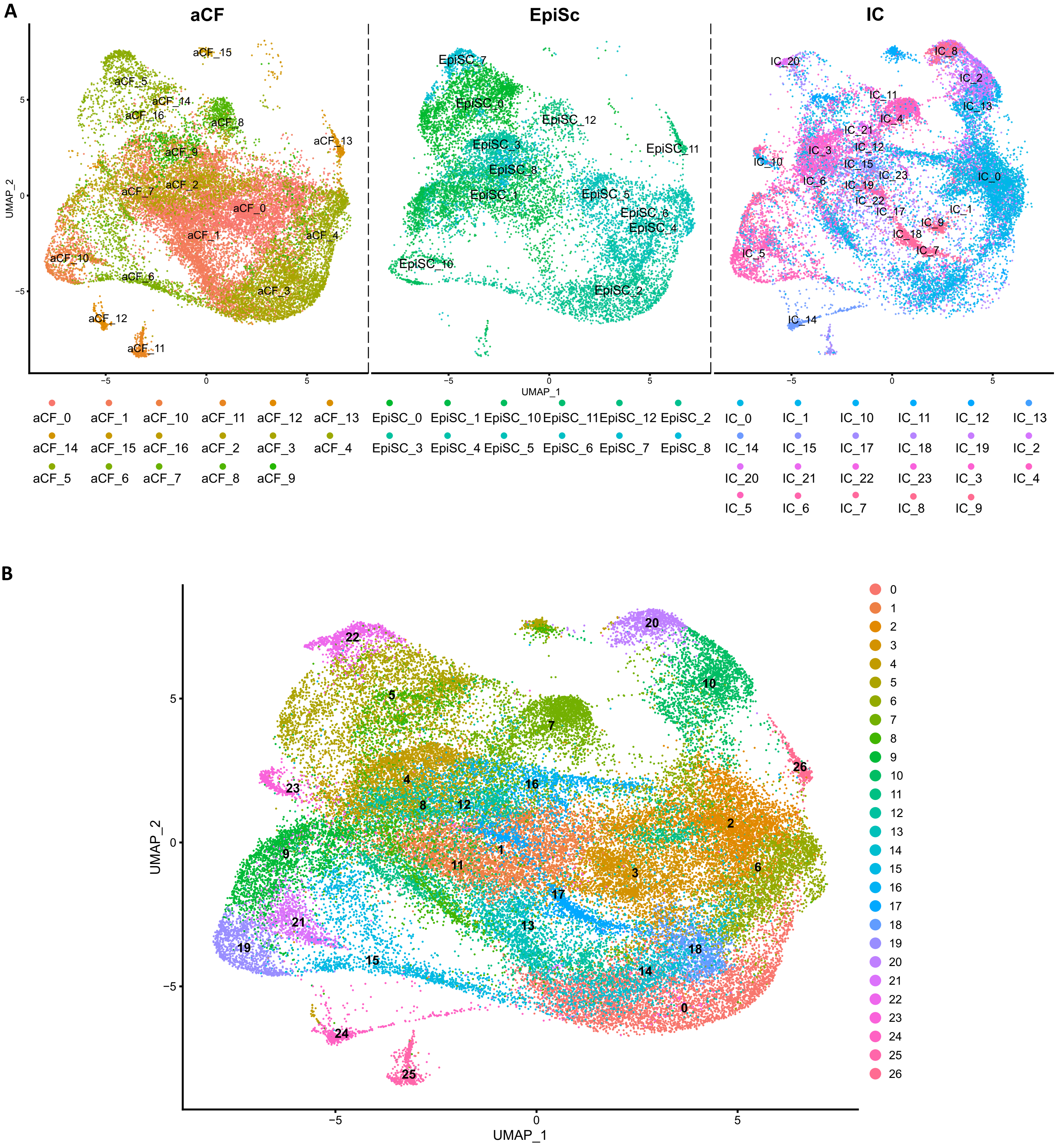

Supplemental Figure S1: Cell populations of activated cardiac fibroblasts (aCF), epicardial stromal cells (EpiSC) and immune cells (IC) from the infarcted heart: Single cell expression profiling of cardiac cells from murine hearts (n=3) was performed on day 5 after infarction (50 min ischemia/reperfusion). Hearts were processed as described earlier[21]to obtain epicardial cells (EpiSC, sorted for $CD31^-CD45^-$), cardiac fibroblasts (aCF sorted for $CD31^-$ $CD45^-$) and cardiac immune cells (IC, CD45, sorted $CD31^-CD45^+$) from the same heart. (**A**) Cell preparations (EpiSC, aCF and IC) were analyzed by scRNAseq in triplicate using the 10x Genomics Chromium platform. Data for aCF and EpiSC were recently published[22].(**B**) combined cell clusters from aCF, EpiSC and IC revealing 26 cell clusters altogether. Unbiased clustering using the Seurat R package with visualization in uniform manifold approximation and projection (UMAP) dimension reduction plots was performed to identify cells with distinct lineage identities and transcriptional similarities. EpiSC: epicardium derived stromal cells; aCF: activated cardiac fibroblasts; IC: immune

Supplemental Figure S2

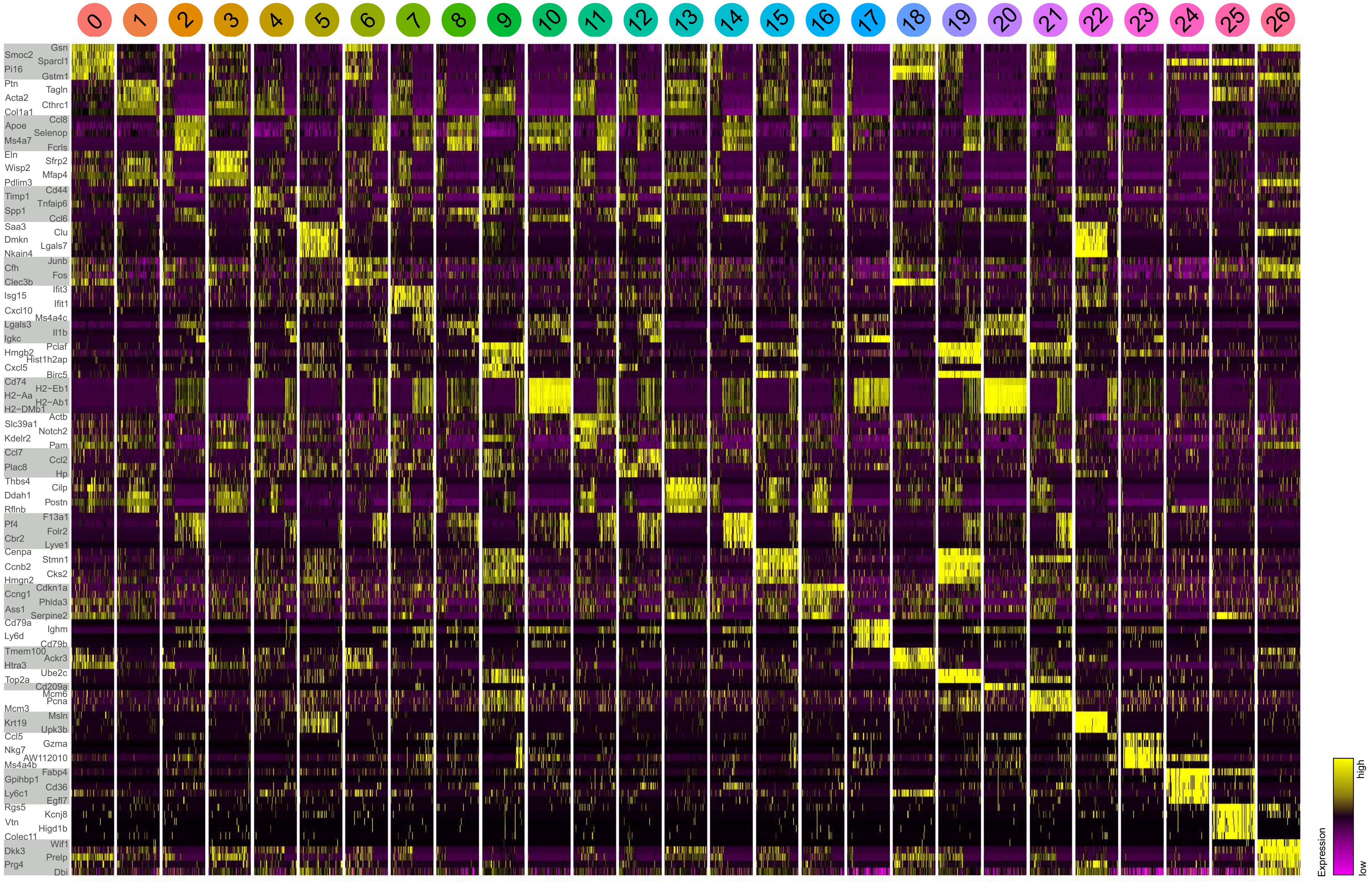

Supplemental Figure S2: Heatmap of the top five marker genes from combined clusters shown in Figure S1: Numbers and colors indicate cluster affiliation.

Supplemental Figure S3

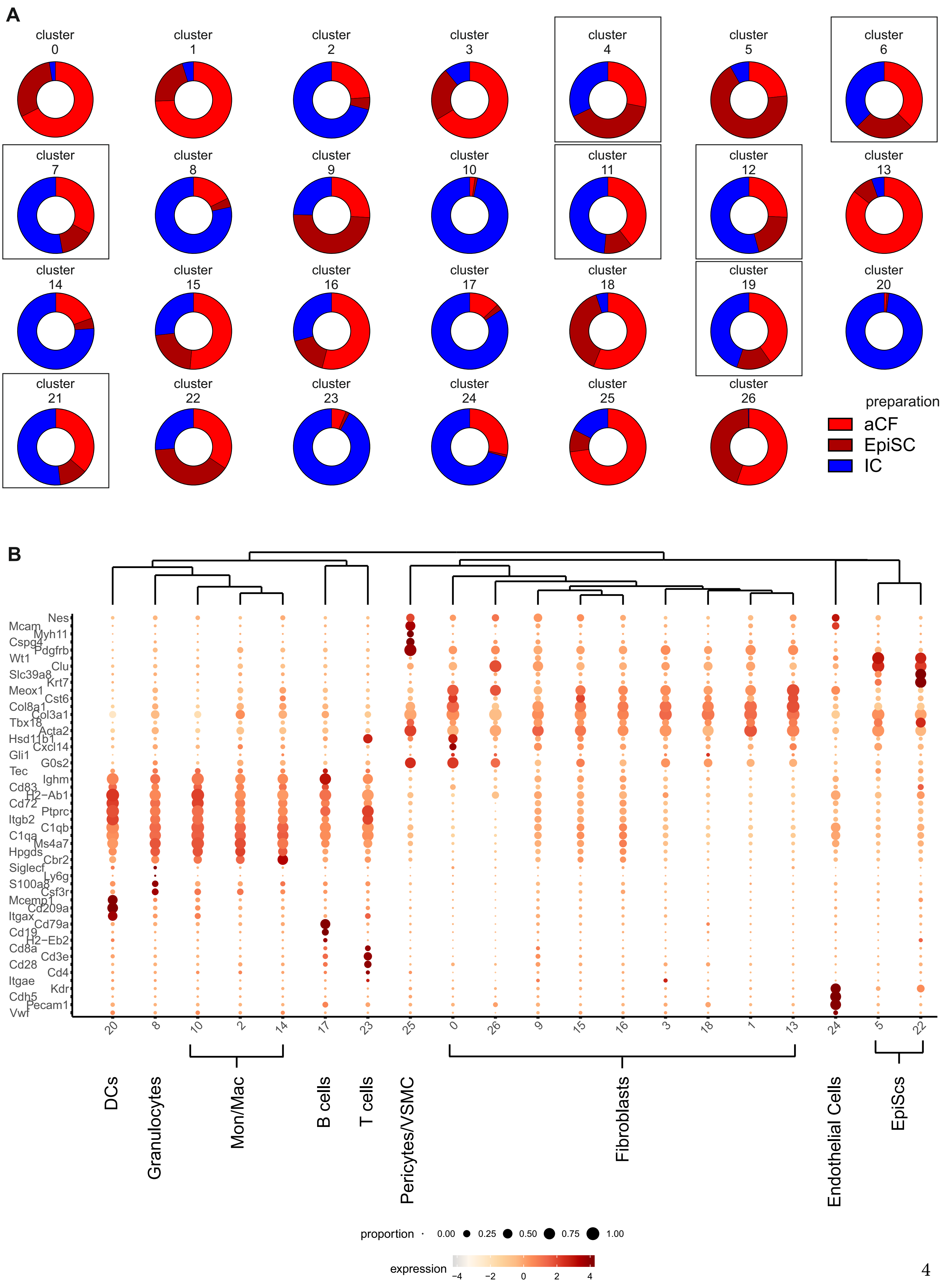

Supplemental Figure S3: Characterization of clusters from combined analysis. (**A**) fractional contribution of aCF, EpiSC and IC within each of the combined 26 clusters shown in fig. S1 and S2. Clusters with no clear affiliation to either immune cells or stromal cells (EpiSC, aCF) are box marked and were excluded from further analysis because of no stringent cell type affiliation. (**B**) Dot-plot representation of marker genes for cell type characterization of individual clusters. EpiSC: epicardium derived stromal cells; aCF: activated cardiac fibroblasts; IC: immune cells.

Supplemental Figure S4

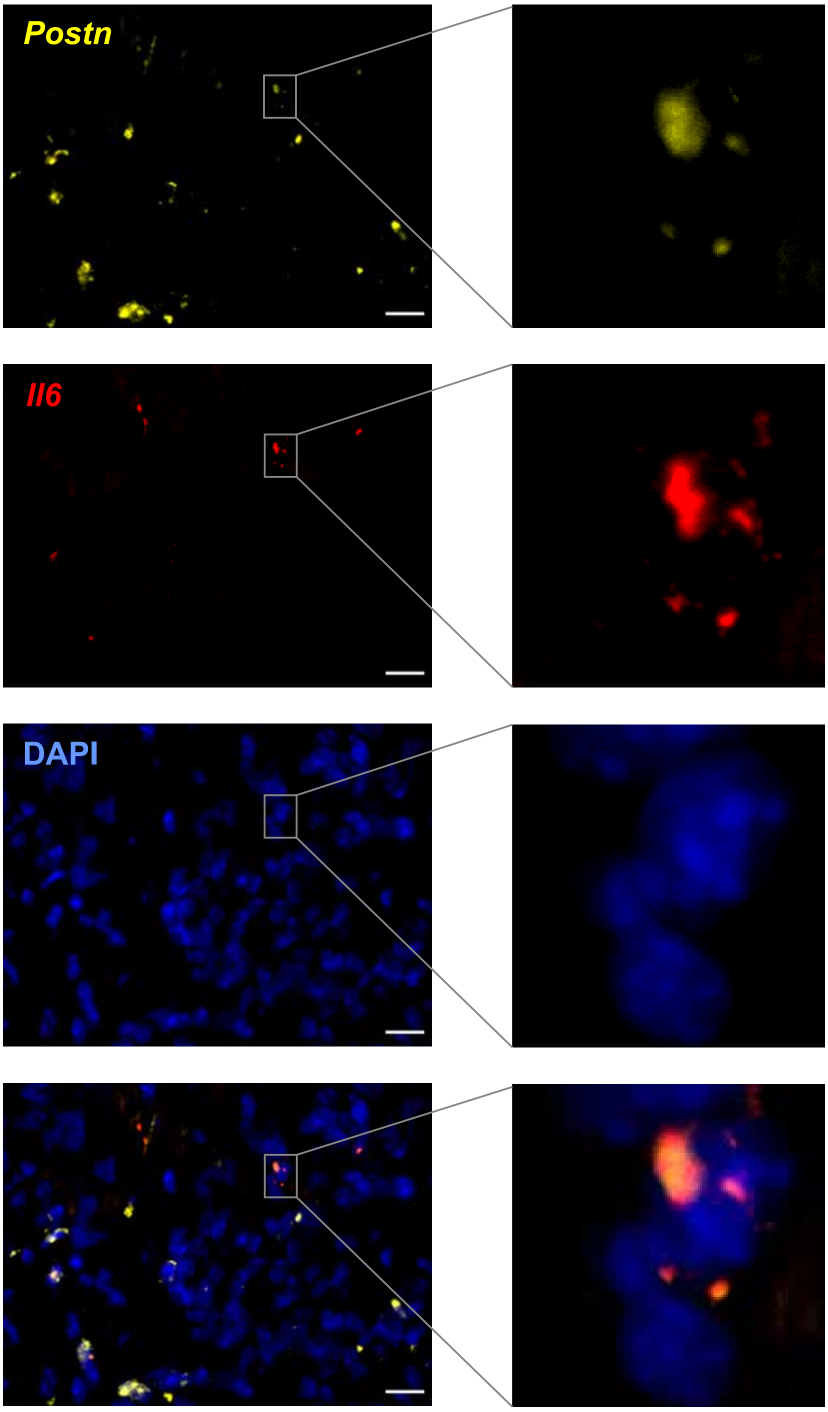

Figure S 4: *Il6* expression in the infarcted heart as measured by RNAscope: Sections from the heart were analyzed three days post MI. Left: representative pictures of the border zone of the left ventricular wall. Fluorophore -labeled probes specific for *Postn* (yellow) and *Il6* (red) were used and nuclei were counterstained with DAPI. Scale bar: 20 µm. Right: close ups enlarged from the corresponding picture on the left. Enlarged area is marked by a grey box.

Supplemental Figure S5

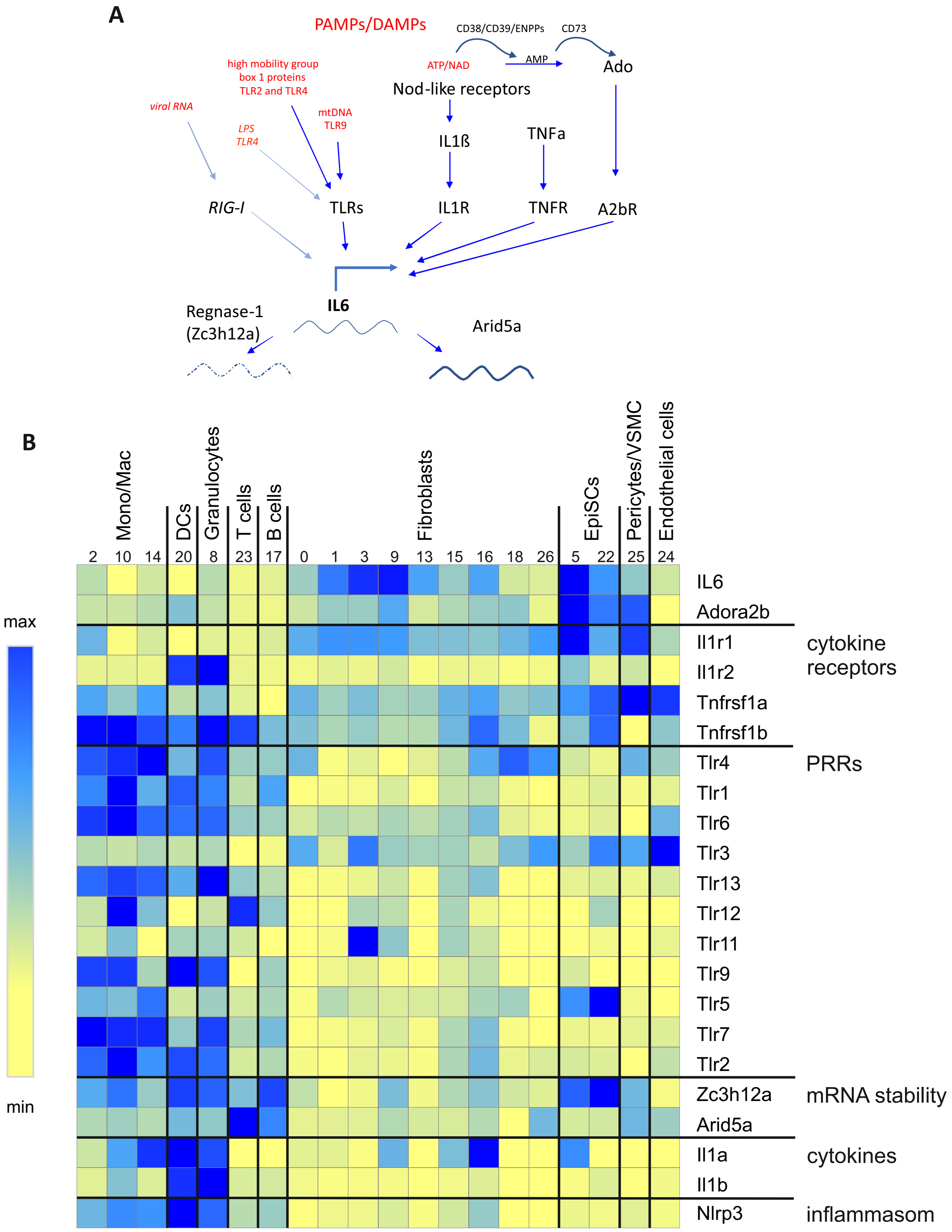

Supplemental Figure S5: Regulation of *Il6* expression: **A** Scheme of molecular interaction to induce Il6 expression or regulate Il6 mRNA stability. While lipopolysaccharides (via Toll-like receptor (TLR4)) and viral RNA (via retinoic acid inducible gene I (RIG-I)) are strong inducers of IL-6 those pathways are supposed to be neglectable in the course of the sterile inflammation that follows after myocardial infarction. In this setting, DAMPs like mitochondrial DNA (TLR9) or high mobility box 1 proteins (TLR2 and 4) can directly induce *Il6* expression. Others like ATP or NAD can induce Nod-like receptor expression and Nlrp3 inflammasome assembly that leads to IL1β secretion which in turn, like TNF-α, can induce *Il6* expression after activating its receptor. However, ATP and NAD are rapidly degraded by an ecto-enzyme cascade involving CD38 (NAD degradation), CD39 and pyrophosphatases (ENPPs) (ATP and/or ADP degradation) and CD73 (AMP degradation) to generate adenosine which was reported to induce IL-6 via A2bR. Additionally, Il6 mRNA can be stabilized by the Arid5a activity or destabilized by Regnase-1 activity. Modified and visualized according to doi: 10.1101/cshperspect.a028456). **B**. Heat map visualizing the expression patterns of several *Il6* regulating factors as obtained by the single cell RNA sequencing analysis introduced in Figure S1-S3. Colum numbers represent gene clusters, colum lettering described cellular identity of cluster groups and row lettering indicates the gene symbol of the corresponding. Heat map was generated using Morpheus, https://software.broadinstitute.org/morpheus. PRRs: pattern recognition receptors

Supplemental Figure S6

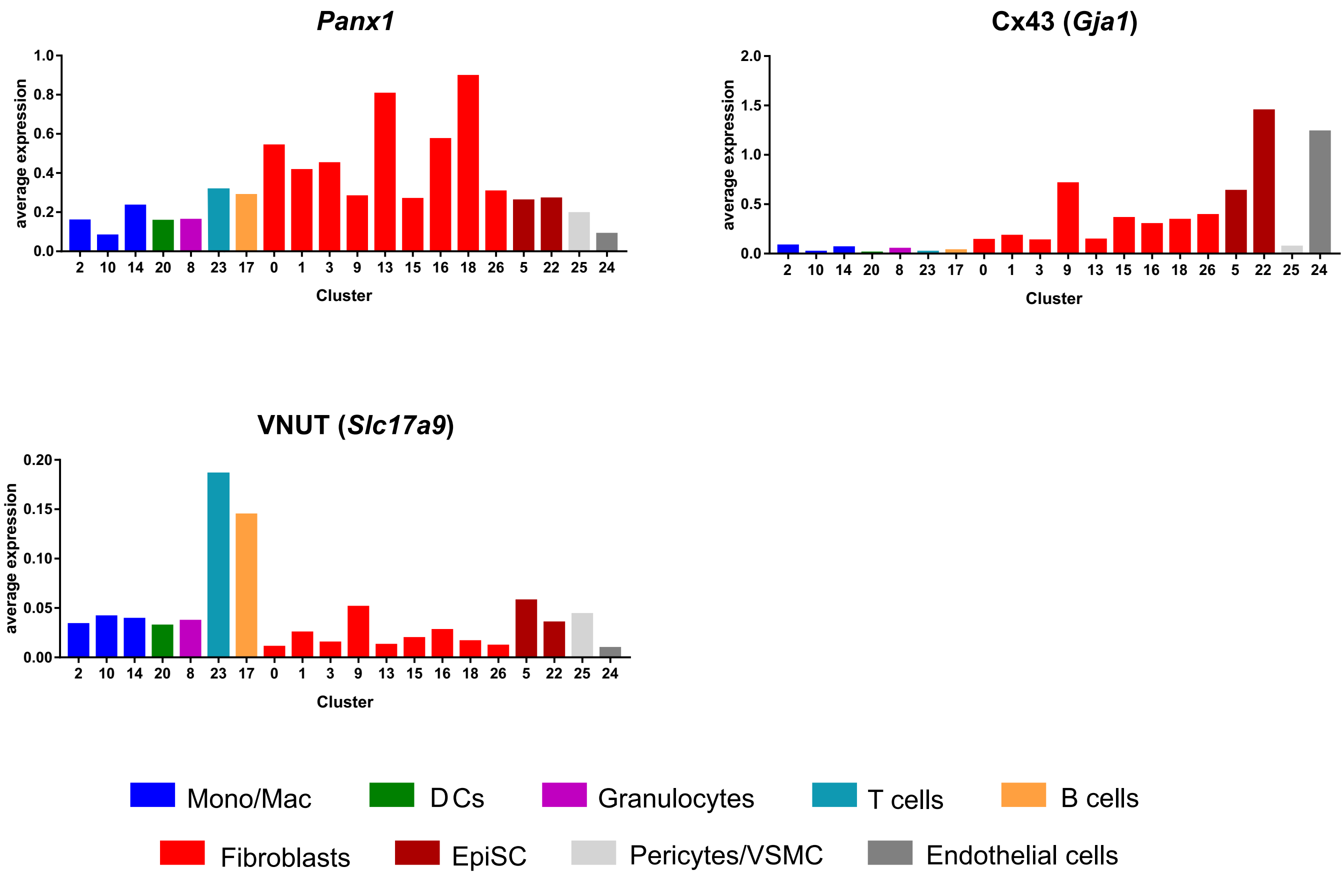

Supplemental Figure S6: Cellular distribution of Panx1, Cx43 and VNUT: Expression of genes coding for putative ATP-transporting proteins *Panx1*, Cx43 (*Gja1*) and VNUT (*Slc17a9*) within the combined cell clustes of aCF, EpisC and immune cells on day 5 post MI. Cell type affiliation is color-

Supplemental Figure S7

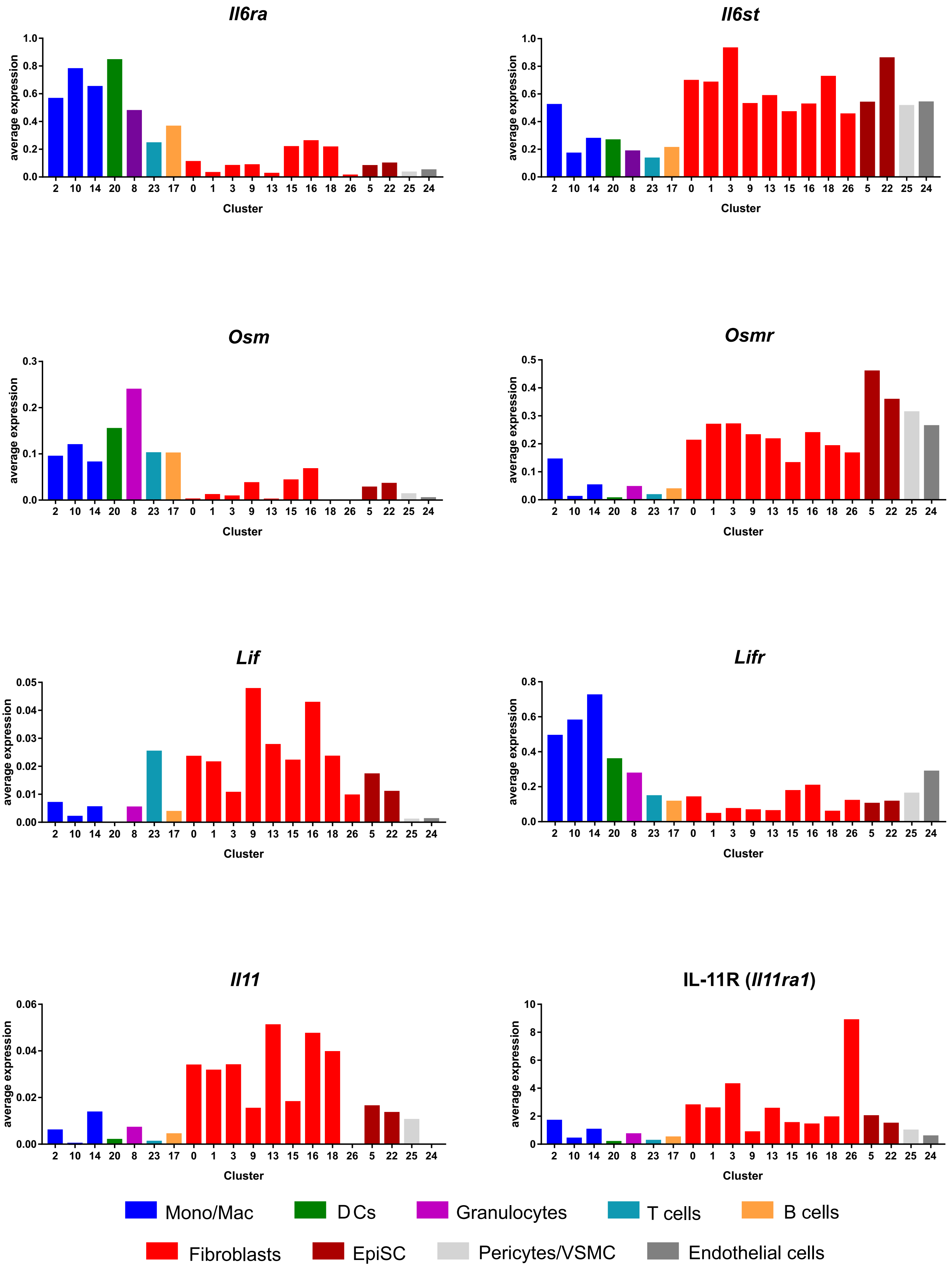

Supplemental Figure S7: Cellular distribution of *Il6Ra, Il6st, Osm*, *Lif* and *Il11* and their respective receptors: Expression of the IL6-family cytokines and their receptor as distributed within the combined cell clustes of aCF, EpisC and immune cells on day 5 post MI. Cell type affiliation is color-coded (n=3).

Supplemental Figure S8

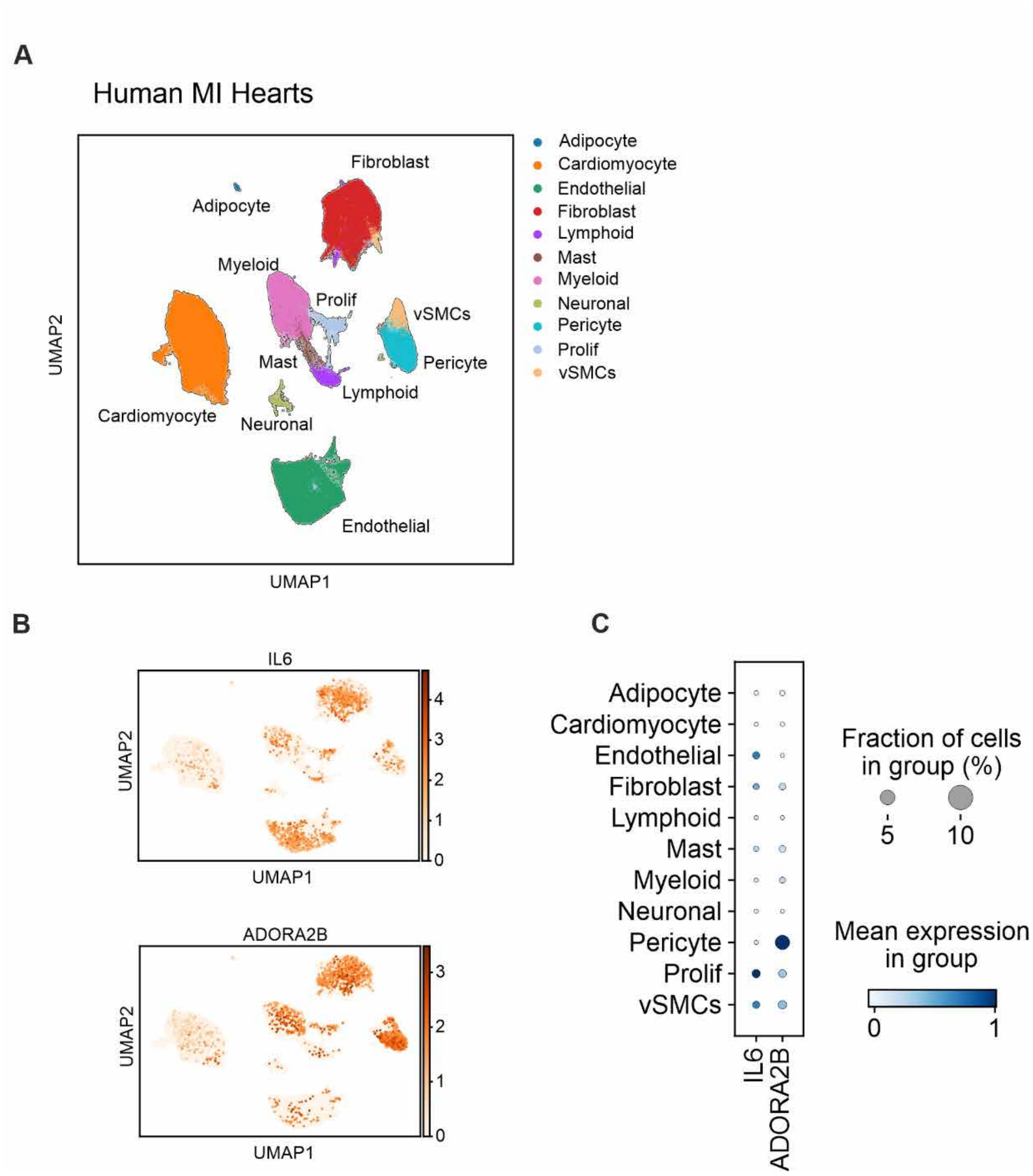

Supplemental Figure S8: IL6 and ADORA2B expression in different human cardiac cell types obtained by single nucleus RNA sequencing (snRNAseq) post MI: Data are from Kuppe et al. 2022 (33) who report multi-omic analyses of 31 cardiac samples of human explanted hearts from MI patients and controls ranging from 0 to 170 days after onset of clinical symptoms. Samples are taken from border zone, remote zone, ischemic zone and fibrotic zone. Kuppe et al. kindly provided the IL6 and ADORA2B expression data obtained by snRNAseq. **A**: UMAP representation and cell-type annotation of 191795 cells obtained from 31 human samples (23 patients) **B**: UMAP representations of cells expressing IL6 or ADORA2B respectively, cell-type annotation corresponds to that in A. **C**: Dot plot representation of IL6 and ADORA2B expression: number of positive cells per cell type is represented by the diameter of the circles, mean expression values are represented by the color intensity.

Supplemental Figure S9

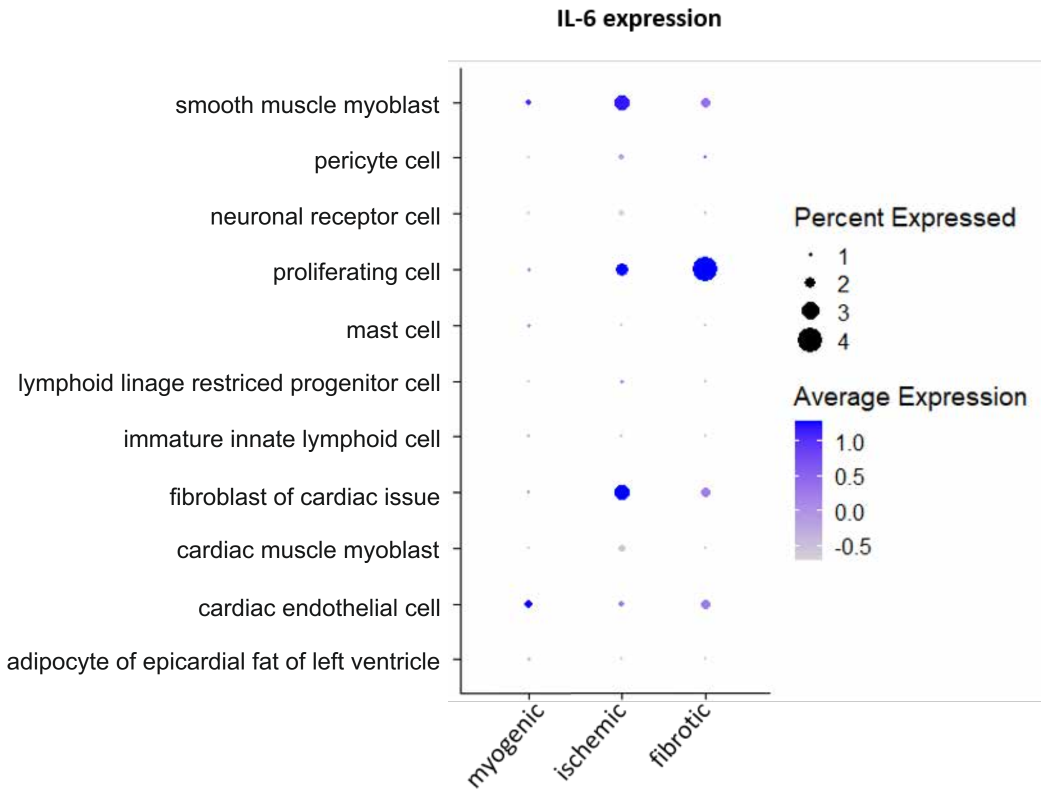

Supplemental Figure S9: IL6 expression in different human cardiac cell types obtained by single nucleus RNA sequencing (snRNAseq). Data are from healthy tissue (myogenic), from acutely infracted tissue (ischemic) and chronically fibrotic tissue (fibrotic). Dot plot representation of IL6, number of positive cells per cell type is represented by the diameter of the circles, mean expression values are represented by the color intensity. Data are from Kuppe et al. 2022 (**33**) who reported multi-omic analyses of 31 cardiac samples of human explanted hearts from MI patients and controls ranging from 0 to 170 days after onset of clinical symptoms.

Supplemental Figure S10

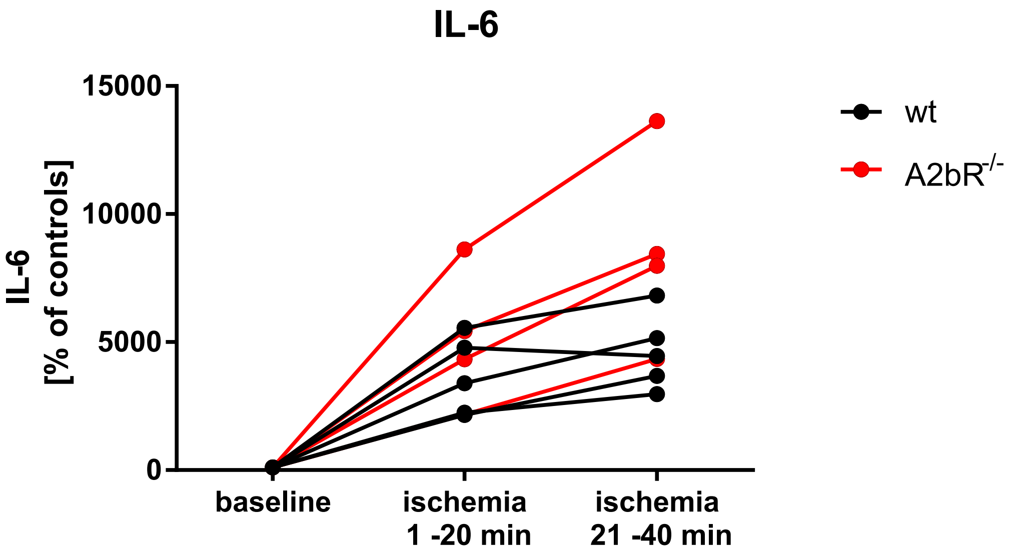

Supplemental Figure S10: IL-6 released into the coronary effluent perfusate of isolated saline perfused hearts (Langendorff) subjected to 30 min ischemia. Perfusates were collected from hearts of WT and A2bR$^{-/-}$ mice over 20 min at baseline and between min 1-20 and min 21-40 post ischemia. Coronary perfusates were concentrated using 10 kDa centrifugation filters and IL-6 was measured by Bioplex. WT n=5, A2bR$^{-/-}$ n=4.

Supplemental Table S1

Supplemental Table S1: Purinergic metabolits released into the coronary effluent perfusate of isolated saline perfused hearts (Langendorff) subjected to 30 min ischemia. Perfusates were collected from WT mice at baseline and at 1, 10 and 30 min post ischemia. Purinergic metabolits concentration was measured by HPLC directly from unconcentrated aliquots. n=5.

µM	ATP mean ±SD	ADP mean ±SD	AMP mean ±SD	adenosine mean ±SD	inosine mean ±SD
normoxic basline	0.23 ±0.34	0.18 ±0.20	0.05 ±0.05	0.31 ±0.46	0.34 ±0.44
1 min post ischemia	11.25 ±1.33	7.92 ±1.36	0.25 ±0.08	5.22 ±2.99	26.32 ±0.92
10 min post ischemia	1.47 ±0.39	1.39 ±0.12	0.02 ±0.01	1.33 ±0.63	3.20 ±0.84
30 min post ischemia	0.62 ±0.30	0.64 ±0.25	0.01 ±0.01	0.28 ±0.15	0.81 ±0.32
